# Anions Containing Tripoid Conjugated N_4_^−^ System: Salts of 5-(Substituted Amino)-[1,2,3]triazolo[4,5-*c*][1,2,5]oxadiazol-5-ium-4-ides, as well as Their Synthesis, Structure, and Thermal Stability

**DOI:** 10.3390/molecules27196287

**Published:** 2022-09-23

**Authors:** Alexey A. Voronin, Sofya P. Balabanova, Ivan V. Fedyanin, Aleksandr M. Churakov, Alla N. Pivkina, Yurii A. Strelenko, Michael S. Klenov, Vladimir A. Tartakovsky

**Affiliations:** 1 N.D. Zelinsky Institute of Organic Chemistry, Russian Academy of Sciences, 47 Leninsky Prosp., 119991 Moscow, Russia; 2 A.N. Nesmeyanov Institute of Organoelement Compounds, Russian Academy of Sciences, 28 Vavilova St., 119991 Moscow, Russia; 3 N.N. Semenov Federal Research Center for Chemical Physics, Russian Academy of Sciences, 4 Kosygina St., Building 1, 119991 Moscow, Russia

**Keywords:** heterocycles, 1,2,5-oxadiazoles, 1,2,3-triazoles, triazenes, diazonium, NMR, X-ray

## Abstract

A strategy for the synthesis of 5-((2-cyanoethyl)-X-amino)-[1,2,3]triazolo[4,5-*c*][1,2,5]oxadiazol-5-ium-4-ides (X = H; CH_2_CH_2_CN; NO_2_ (**4a**); CN (**4b**); CO_2_Et (**4c**)) starting from 3-amino-4-azido-1,2,5-oxadiazole was developed. The key step in this strategy is the intramolecular thermolytic cyclization of the azido group and the bis(2-cyanoethyl)triazene group. Removal of the 2-cyanoethyl protecting group from amides **4a**–**c** gave potassium salt of the corresponding nitramide and sodium salts of cyano- and ethoxycarbonylamide. The structure and thermal stability of the synthesized compounds were studied experimentally using multinuclear NMR spectroscopy, X-ray crystallography, thermogravimetry, and differential scanning calorimetry.

## 1. Introduction

**Figure 1:**
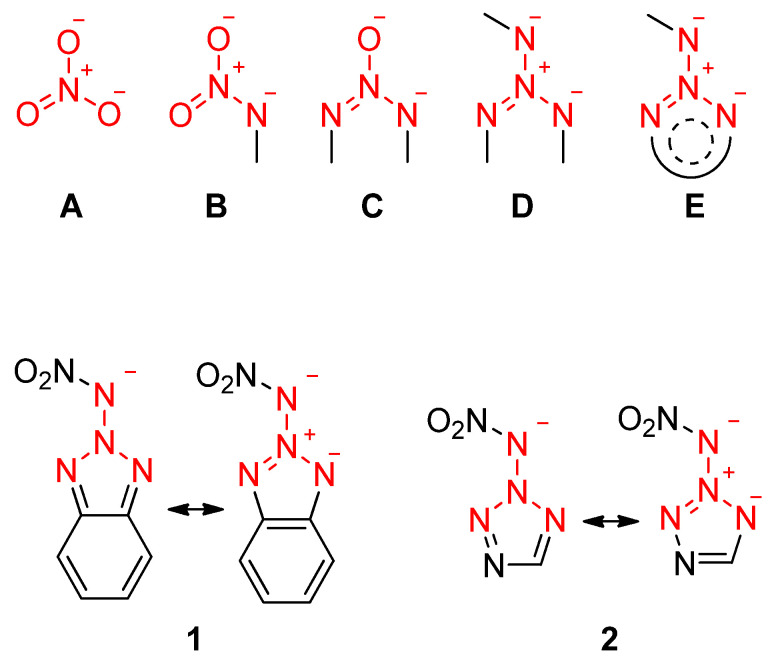
Nitrate anion **A**, related isoelectronic structures **B**–**E**, and **E**-type anions **1** and **2**.

Nitrate anion **A** has a high thermal stability. Sodium and potassium nitrates begin to decompose at temperatures above 380 °C. Chemists are faced with the task of studying the changes in the thermal stability of compounds when oxygen atoms in the nitrate anion are replaced by nitrogen atoms (Figure 1).

The structure of the nitrate anion has the following features: (1) a flat Y-shaped (tripoid) topology; (2) the positive charge is localized on the central N-atom of the system, and the negative charges are distributed over three terminal O-atoms; and (3) six electrons take part in conjugation (the so-called Y-aromaticity [1]).

With the successive isoelectronic substitution of oxygen atoms in the nitrate anion **A** for nitrogen atoms, anions **B**, **C,** and **D** are formed, while the conjugated 6-π electron system is retained (Figure 1). In the case when substituents in the nitrogen atoms exhibit the –M effect (NO_2_, CN, etc.), negative charge delocalization is possible, which can lead to an elongation of the conjugation chain and a probable increase in the stability of the electron shell of the system.

Representative compounds of type **B** are the well-known anions of primary nitramines. Compounds of type **C** are represented in the literature as anions of unannulated and annulated 1,2,3-triazole 2-oxides and tetrazole 2-oxides. Type **D** acyclic anions are not described (for theoretical studies of such systems, see [2]), and type **E** anions are represented by compounds **1** [3] and **2** [4], in which three of the four atoms of the tripoid conjugated system are built into a five-membered heterocyclic ring (Figure 1).

Note that the thermal stability of compounds **B**–**E** may decrease compared with the nitrate anion, as the substituents may give rise to new pathways for the decomposition of the molecule, which were not present in the nitrate anion. An example of **B** compounds that are less thermally stable than the nitrate anion are dinitramide salts [5,6,7].

Previously, our group systematically studied the compounds of type **B** (dinitramide salts [8,9]) and **C** (4*H*-[1,2,3]triazolo[4,5-*c*][1,2,5]oxadiazole 5-oxide [10]). Now, we are starting to study the compounds of type **E**.

**Figure 2:**
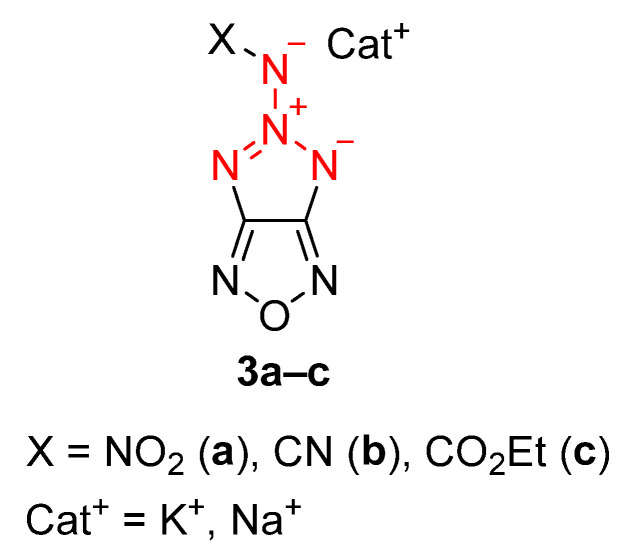
Compounds described in this paper.

This article describes an approach to the synthesis of salts **3a**–**c** and investigates their structure and thermal stability (Figure 2).

## 2. Results and Discussion

### 2.1. Synthesis

**Scheme 1:**
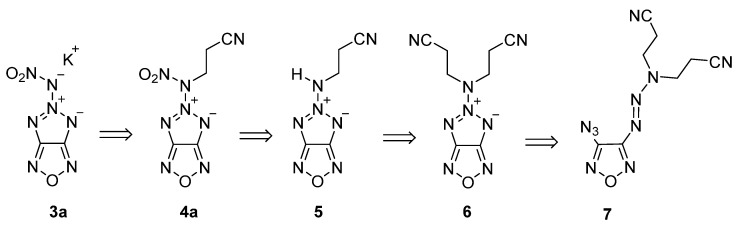
Retrosynthetic analysis of salt **3a**.

We started our study with compound **3a**. Typically, *N*-nitramides of heterocycles are prepared through nitration of the appropriate *N*-amino heterocycles (for review, see [11]). However, sometimes, these compounds are not available. We developed an alternative approach to the synthesis of target salts (Scheme 1). The main idea in this route is the synthesis of compound **6** bearing two cyanoethyl groups and the subsequent stepwise removal of these groups.

Choosing this synthetic route, we kept in mind a number of known reactions. The first one was thermolytic cyclization of 3-azido-4-azo-1,2,5-oxadiazoles leading to 5-Ar(Alk)-substituted 1,2,3-triazolo[4,5-*c*][1,2,5]oxadiazoles [12,13,14,15]. This reaction proceeded smoothly with a good yield when the starting 1,2,5-oxadiazoles (furazans) were heated in organic solvents (acetonitrile, benzene, etc.) at 80 °C for several hours. Related cyclization of the adjacent azido and triazene groups took place in more drastic conditions at temperatures ≥ 130 °C in order to give *N*-substituted 2-aminobenzotriazoles [16,17].

The 2-cyanoethyl group is a well-known protecting group. An example of the use of this group is the synthesis of dinitramide from 3-(dinitroamino)propanenitrile [8,18].

**Scheme 2:**
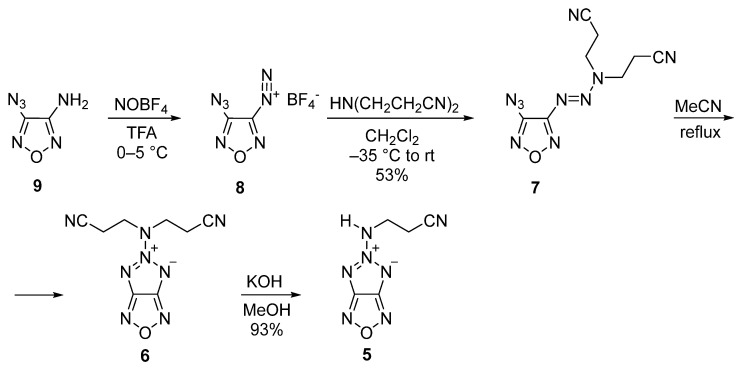
Synthesis of compounds **5**–**7**.

The key compound in our synthetic route, triazene **7**, was obtained through a reaction of diazonium salt **8** with 3,3′-iminodipropionitrile in CH_2_Cl_2_ at –35 °C in a 53% yield (Scheme 2). This salt is a rather labile compound. It was obtained through a modified method that was recently described [19]. Our method involved the reaction of 3-amino-4-azido-1,2,5-oxadiazole **9** with NOBF_4_ in trifluoroacetic acid at 0–5 °C, followed by solvent removal at this temperature in vacuo and immediate cooling to −35 °C. Unlike previous methods for the in situ preparation of salt **8** (*cf*. lit. [14,20]), our method made it possible to isolate diazonium salt **8** in the solid state and to carry out the reaction of this salt with amine in organic solvents.

**Scheme 3:**
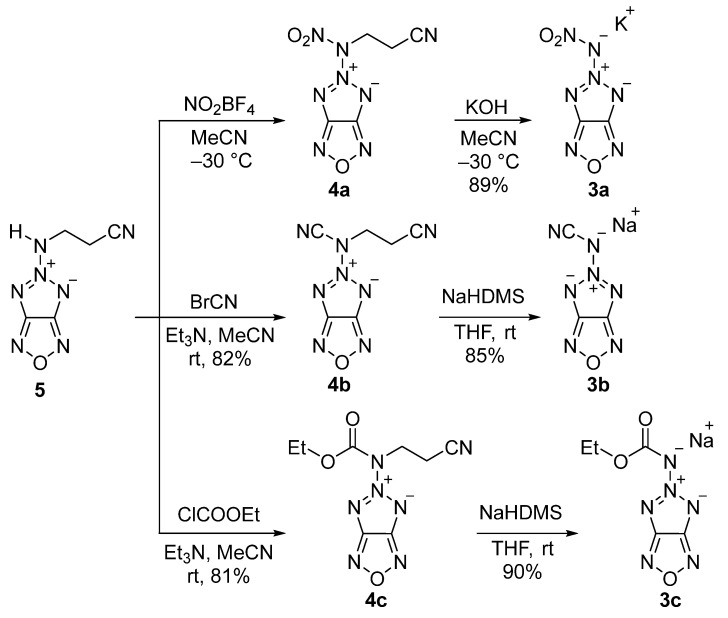
Synthesis of amines **4a**–**c** and amides **3a**–**c**.

The key step in the synthesis of salts **3a**–**c** (Scheme 3) includes an intramolecular reaction of the triazene group and the azido group (Scheme 2). In the 1,2,5-oxadiazole (furazan) series, such cyclization was not previously described. To find the conditions for cyclization, triazene **7** was analyzed by TG-DSC (for details see the “Thermal Behavior” section). As a result, the optimum reaction temperature was found. Refluxing triazene **7** in acetonitrile for 24 h afforded (bis(2-cyanoethyl)amino)triazolofurazan **6** in a quantitative yield. The next step involved the removal of the cyanoethyl group by treating triazolofurazan **6** with a solution of KOH in methanol to provide (cyanoethylamino)triazolofurazan **5** in a 93% yield (Scheme 2).

Scheme 3 shows the synthesis of nitramide **3a**, cyanamide **3b**, and ethoxycarbonylamide **3c**. The nitration of amine **5** with NO_2_BF_4_ at −30 °C in MeCN gave nitro derivative **4a,** which, without isolation, was treated with a solution of KOH (3.2 eq. excess) in MeOH to give K-salt of nitramide **3a**. This salt formed a fairly strong complex with MeCN at a ratio of 1:1, which did not disintegrate during column chromatography (ethyl acetate/methanol, 5:1). The complex completely lost MeCN at 80 °C under reduced pressure within 2 h.

The cyanation of amine **5** with BrCN in the presence of Et_3_N in MeCN gave the *N*-cyano derivative **4b** in an 82% yield. The following treatment of the latter with 1 eq. of sodium bis(trimethylsilyl)amide (NaHMDS, 2M THF solution) in dry THF resulted in the elimination of the cyanoethyl group to give Na-salt of cyanamide **3b**. Purification (flash chromatography, ethyl acetate/methanol, 5:1) gave the dihydrate salt in an 85% yield.

Na-salt of ethoxycarbonylamide **3c** was synthesized in a similar way. First, amine **5** was introduced into the reaction with ethyl chloroformate in the presence of Et_3_N in MeCN, yielding *N*-ethoxycarbonyl derivative **4c** (81%). The latter was treated with 1 eq. of NaHMDS (2M THF solution) in dry THF, resulting in the desired Na-salt of cyanamide **3c** in a 90% yield. Flash chromatography (ethyl acetate/methanol, 5:1) afforded monohydrate of the salt in a 90% yield.

### 2.2. Spectroscopy

All of the compounds obtained were fully characterized by IR (KBr) and multinuclear (^1^H, ^13^C, and ^14^N) NMR spectroscopy recorded in [D_6_]-acetone, [D_6_]-DMSO, or [D_4_]-methanol (for details, see Supplementary Materials).

**Figure 3:**
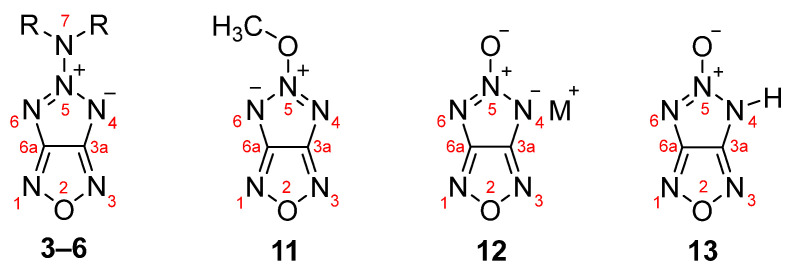
Compounds **11**–**13**.

The cyanoethyl substituents in triazene **7** are non-equivalent and appear in ^1^H NMR spectra as two sets of signals, *δ* = 3.03, 3.15 (t, *J* = 6.7 Hz), 4.29, 4.39 (t, *J* = 6.7 Hz) ppm, which is apparently due to hindered internal rotation about the single N–N bond in the triazene moiety [21]. This phenomenon disappears in the case of bis(2-cyanoethyl)amino)triazolofurazan **6** in the ^1^H NMR spectra, of which only one pair of triplets referring to both cyanoethyl substituents is observed: *δ* = 3.21 (t, *J* = 6.7 Hz), 4.62 (t, *J* = 6.7 Hz) ppm. The same is true for the ^13^C NMR spectra of this compounds. Because of the symmetry of the triazolofurazan moiety in compounds **3**–**6**, atoms C-3a and C-6a are equivalent and appear in the ^13^C NMR spectra as one peak: *δ* = 163±1 ppm (regardless of the solvent). This is quite close to the ^13^C NMR spectra of compounds **11** (C-3a,6a: *δ* = 160.5 ppm) [22] and salts **12** (C-3a,6a: *δ* ~ 161 ppm) [10] (Figure 3).

In all ^14^N NMR [23] spectra of triazolofurazans **3**–**6**, at least two signals related to the heterocyclic framework are observed. One narrow signal of the N-5 atom is in the range from −30 to −80 ppm (Δν_½_ = 60–200 Hz). The exception is the spectrum of compound **4c** in [D_6_]-acetone, in which, unexpectedly, all signals are significantly widened, including the signal of the N-5 atom (*δ* = −77 ppm, Δν_½_ = 770 Hz). Interestingly, in the spectra of the covalent compounds **11** (in CDCl_3_) and **13** (in [D_6_]-acetone), similar signals appear in the same range (*δ* = −50 and −39 ppm, respectively [10,22]).

For compound **6**, ^1^H–^15^N HSQC was performed in [D_6_]-acetone to determine definitely the N-5 signal (*δ* = −52 ppm). It is noteworthy that for compound **5,** the N-5 peak is observed in the same place. Replacing hydrogen with a strong electron withdrawing group (CN, COOEt) in compounds **4b**,**c** results in shifting of the N-5 signal to a stronger field (*δ* = −77 ppm). Similar signals in the case of Na-salts **3b**,**c,** on the contrary, are shifted to a weaker field (*δ* = −31 ppm). At the same time, the N-5 peak of the K-salt of nitramide **3a** is observed in the same range (*δ* = −47 ppm) as in compounds **5** and **6**.

Another type of signal related to the heterocyclic framework is the broadened ones, which could be attributed to N-1 and N-3 of the furazan cycle (*δ* = 0–40 ppm, Δν_½_ = 900–2000 Hz). In the ^15^N NMR spectrum of the nitramide **3a** complex with MeCN in [D_6_]-acetone, a full set of signals is observed: *δ* = 23 (N-1 and N-3), −8 (NO_2_), −49 (N-5), −87, −94 (N-4, N-6, **N**–NO_2_).

### 2.3. X-ray Structure Analysis

**Figure 4:**
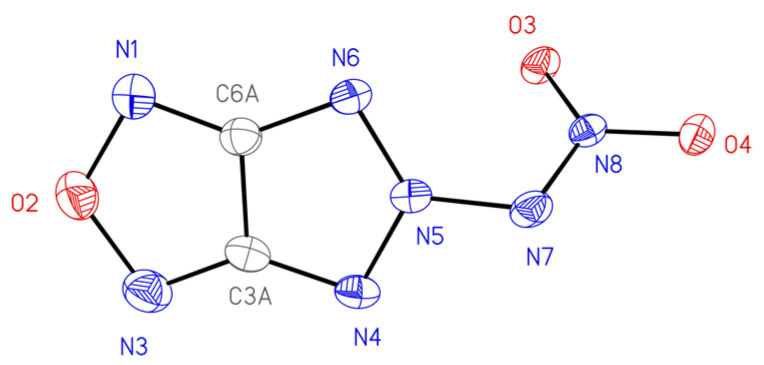
General view of the anion **3a** in crystal. Anisotropic displacement parameters are drawn at a 50% probability level.

The structures of the salts **3a** (in the form of acetonitrile solvate), **3b** (in the form of dihydrate), and **3c** (in the form of monohydrate), as well as molecules **4b**, **6,** and **7** were confirmed using single-crystal X-ray crystallography. As an example, the typical view of the anion **3a** is provided in Figure 4.

In all of the molecules obtained, all atoms of the triazolofurazan framework, together with the exocyclic N7 atom, lie in the same plane. Both in anions **3a**–**c** and in neutral molecules **4b** and **6**, the corresponding bond lengths of the furazan ring are almost the same (for details see Supplementary Materials).

**Figure 5:**
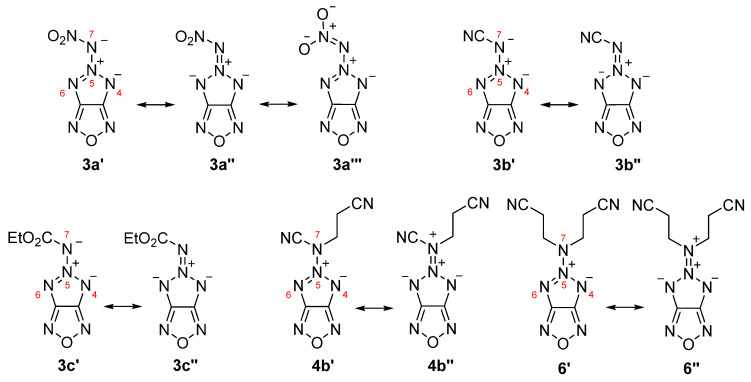
Resonance structures of compounds **3a**–**c, 4b**, and **6**.

The compounds studied by X-ray diffraction analysis can be divided into two groups. The first group includes anions **3b** and **3c** and (bis(2-cyanoethyl)amino)triazolofurazan **6**. The second group includes nitro-substituted anion **3a** and cyano-substituted compound **4b** (Figure 5).

In all of the compounds of the first group, an effective transfer of a negative charge from the N7 atom to the triazole ring is observed. As a result, the N5–N7 exocyclic bond, and the N4–N5 and N5–N6 endocyclic bonds of the tripoid system are aligned (*ca*. 1.35 Å) (Table 1). In terms of the resonance theory, this means a large contribution from the resonance structures **3b"**, **3c"**, and **6"** (Figure 5). Note that the effect of the exocyclic oxygen atom in anion **12** on the bond lengths in the triazole ring is practically the same [10].

For compounds of the second group, the negative charge transfer from the N7 atom occurs not so much in the triazole ring as in the electron-withdrawing substituent, to the cyano group in compound **4b** and to the nitro group in the anion **3a**. As a result, the N5–N7 bond becomes longer (*ca.* 1.38 Å), while the N4–N5 and N5–N6 bonds are somewhat shorten (*ca.* 1.33 Å). In terms of the resonance theory, this means a small contribution from the resonance structures **3a"** and **4b"**(Figure 5).

It is important to note that in the case of amides **3b** and **3c**, the furazanotriazole ring is practically coplanar with the exocyclic substituents N–CN and N–COOEt, i.e., the substituents and the ring form a single conjugated system. In nitramide **3a**, the furazanotriazole ring and the N–NO 2 fragment are not coplanar, with a dihedral angle of 58.2° to the ring plane. A similar situation was observed in the case of pyridine-1-nitramide, for which the dihedral angle between the N-NO 2 fragment and the pyridine ring is 71.7° [24]. Thus, in nitramide **3a**, the furazanotriazole ring and the nitro group do not form a single conjugated system.

### 2.4. Thermal Behavior

The thermal stability of the investigated compounds was assessed by tracking the signals of the thermogravimetry (TG) and differential scanning calorimetry (DSC) (for detail see Supplementary Materials). As a first measure of the thermal stability, the extrapolated onset of the decomposition peak can be used. Note that a more precise conclusion can be drawn only after kinetic analysis of the decomposition process [25]. Through the extrapolated onsets of the decomposition peak, the thermal stability of the analyzed compounds increased in a row, as follows: **5** (127 °C) < **3a** (152 °C) ≈ **3c** (165 °C) < **4b** (185 °C) ≈ **3b** (190 °C) < **4c** (233 °C) ≈ **6** (234 °C).

Nitramide **3a** melts at 152 °C with the subsequent decomposition. Cyanamide **3b** upon heating shows a series of weak endothermic events with the marked exotherm of the thermal decomposition with an extrapolated onset of 190 °C. Initial endotherms apparently correspond to the elimination of water, this conclusion is supported by nearly 16% of the mass loss prior to 150 °C. Ethoxycarbonylamide **3c** shows a more complex picture, including an endothermic event that shifts to exothermic events consisting of two peaks: the first peak is 122 °C, the second one is 170 °C. As for **3b**, initial endotherm probably corresponded to the elimination of water. The subsequent exothermic event with an extrapolated onset of 122 °C most likely was not related to the decomposition of the heterocyclic system. This is supported by nearly 10% of mass loss prior to 150 °C. Apparently, the second peak with an extrapolated onset of 170 °C refers to the decomposition of ethoxycarbonylamide **3c**.

Covalent (2-cyanoethyl)amines **4b,c** are thermostable but low-melting compounds. So, compound **4b** melts at 76 °C with a thermal decomposition at further heating (extrapolated onset: 185 °C). Compound **4c** reveals first endothermic event at 61 °C, and then decomposes above 200 °C in these experimental conditions. Bis(2-cyanoethyl)amine **6** melts at 139 °C with the decomposition at further heating (extrapolated onset: 234 °C). As expected, amine **5** bearing the NH proton has one of the lowest thermal stabilities, decomposing right after the endothermic event at 127 °C, as evidenced by the heat release and mass loss signals. The endotherm at 127 °C is preceded by two weaker endothermic events without noticeable mass loss. The tentative interpretation of these effects is a phase transition, and for endotherm at 127 °C, the melting of amine **5**.

It can be seen that both covalent and ionic compounds have a relatively high thermal stability (>150 °C), except for the triazolofurazan **5** bearing NH proton. This suggests that 5-amino-[1,2,3]triazolo[4,5-*c*][1,2,5]oxadiazol-5-ium-4-ide is a promising scaffold for the design of various thermally stable systems.

Triazene **7** at heating first reveals the endotherm at 79 °C, followed by an exothermic event with a maximum at 133 °C, and after 200 °C, a sharp peak of the heat release is observed. The tentative assignment of the first effect on DSC trace (endotherm at 79 °C) is melting. The following exotherm shows the associated mass loss of 11 wt.%, which closely matches the elimination of one molecule of nitrogen from triazene **7**. The last exotherm with an extrapolated onset of 221 °C is thus the thermal decomposition of the formed amine **6**.

## 3. Conclusions

In conclusion, we developed a strategy for the synthesis of *N*-substituted 5-aminotriazolofurazans **3**–**6** starting from 3-amino-4-azido-1,2,5-oxadiazole **9**. Previously unknown in the 1,2,5-oxadiazole series in the intramolecular thermolytic cyclization of the azido group and the bis(2-cyanoethyl)triazene group was investigated. This cyclization opens the door to compounds containing tripoid conjugated system consisting of four nitrogen atoms. The key compound in our synthetic route, triazene **7**, was prepared through the the reaction of 3,3′-iminodipropionitrile with diazonium salt **8** in dichloromethane. The latter salt was synthesized by a method involving the reaction of 3-amino-4-azido-1,2,5-oxadiazole **9** with NOBF_4_ in trifluoroacetic acid, followed by solvent removal to give diazonium salt in the solid state. The structures of the salts **3a**–**c,** as well as covalent compounds **4b**, **6,** and **7,** were confirmed using single-crystal X-ray crystallography. According to the TG-DCS analysis, the extrapolated onset temperatures for the decomposition of compounds **3, 4,** and **6** were between 152 and 234 °C. This indicates a relatively high stability for both covalent and ionic compounds of this type, and allows us to consider 5-amino-[1,2,3]triazolo[4,5-*c*][1,2,5]oxadiazol-5-ium-4-ide as a promising scaffold for creating various thermally stable systems.

## 4. Materials and Methods

CAUTION!!! Although we encountered no difficulties during the preparation and handling of the compounds described in this paper, they are potentially explosive energetic materials that are sensitive to impact and friction. Any manipulations must be carried out using the appropriate standard safety precautions.

**General.** All reactions were carried out in well-cleaned oven-dried glassware with magnetic stirring. ^1^H, ^13^C, ^14^N, and ^15^N NMR spectra were recorded with a Bruker DRX-500 (500.1, 125.8, and 36.1 MHz, respectively) and Bruker AV600 (600.1, 150.9, 43.4, and 60.8 MHz, respectively) spectrometers. Chemical shifts are reported in delta (*δ*) units, parts per million (ppm) downfield from internal TMS (^1^H, ^13^C) or external CH_3_NO_2_ (^14^N, ^15^N negative values of *δ*_N_ correspond to upfield shifts); multiplicities are indicated by s (singlet), d (doublet), t (triplet), q (quartet), m (multiplet), and br (broad). The IR spectra were recorded with a Bruker “ALPHA-T” spectrometer in the range of 400–4000 cm^−1^ (resolution 2 cm^−1^) as pellets with KBr or as a thin layer. Elemental analyses were performed with the CHN Analyzer Perkin-Elmer 2400 or EuroVector EA. High-resolution ESI mass spectra (HRMS) were recorded with a Bruker micrOTOF II instrument. All of the measurements were performed in positive (+MS) or negative (–MS) ion mode (interface capillary voltage: 4500 V) with a scan range of *m*/*z* 50–3000. External calibration of the mass spectrometer was performed with Electrospray Calibrant Solution (Fluka). A direct syringe injection was used for all of the analyzed solutions in MeCN (flow rate: 3 µL·min^−1^). Nitrogen was used as a nebulizer gas (0.4 bar) and dry gas (4.0 L·min^−1^); the interface temperature was set at 180 °C. All of the spectra were processed using the Bruker DataAnalysis 4.0 software package. Thermal analysis was performed with a Netzsch STA 449F1 instrument. Differential scanning calorimetry (DSC) and thermogravimetry (TG) signals were obtained for the sample of ca. 1 mg enclosed in an aluminum crucible with a pierced lid and heated linearly at 5 K·min^−1^ rate in argon flow. Analytical thin-layer chromatography (TLC) was carried out on Merck 25 TLC silica gel 60 F_254_ aluminum sheets. The visualization of the TLC plates was accomplished with a UV light. Silica gel 60 Merck (15–40 μm) was used for the preparative column chromatography. All of the reagents were purchased from Acros or Sigma-Aldrich. The solvents were purified before use, according to standard procedures. 3-Amino-4-azido-1,2,5-oxadiazole **9** was prepared according to a previously published procedure [20].

**(*E*)-3,3’-(3-(4-Azido-1,2,5-oxadiazol-3-yl)triaz-2-ene-1,1-diyl)dipropanenitrile (7)**. NOBF_4_ (760 mg, 6.5 mmol) was added to a vigorously stirred solution of 3-amino-4-azido-1,2,5-oxadiazole **9** (760 mg, 6 mmol) in TFA (4 mL) at 0–5 °C under an argon atmosphere. The reaction mixture was stirred for 3 h, after that it was concentrated under reduced pressure at 0–5 °C. The obtained slurry was cooled to −35 °C, then a cooled (−35 °C) 3,3′-iminodipropionitrile (3 g, 24.4 mmol) solution in CH_2_Cl_2_ (10 mL) was added. The reaction mixture was smoothly warmed to RT under vigorous stirring. The solvent was evaporated in vacuo and the residue was purified by flash chromatography (ethyl acetate/petroleum ether, 1:1) to give a white solid (825 mg, 53%). ^1^H NMR (500.1 MHz, [D_6_]-acetone): *δ* 3.03, 3.15 (t, 4 H, CH_2_C**H**_2_CN, *J* = 6.7 Hz), 4.29, 4.39 (t, 4 H, C**H**_2_CH_2_CN, *J* = 6.7 Hz) ppm. ^13^C NMR (125.8 MHz, [D_6_]-acetone): *δ* 13.1, 16.6 (CH_2_**C**H_2_CN), 43.7, 51.2 (**C**H_2_CH_2_CN), 117.0, 117.1 (CN), 148.1, 154.5 (C(3,4)) ppm. ^14^N NMR (36.1 MHz, [D_6_]-acetone): *δ* –130 (N_3_, ν_½_ = 670 Hz), –144 (N_3_, ν_½_ = 70 Hz). IR (KBr): *ν*˜ 592, 631, 748, 766, 889, 916, 943, 1014, 1146, 1173, 1190, 1279, 1307, 1340, 1349, 1382, 1404, 1438, 1470, 1495, 1561, 2140, 2166, 2254, 2935, 2963, 3012 cm^−1^. HRMS (ESI) *m*/*z* [*M*+NH_4_]^+^ calcd for C_8_H_8_N_10_O: 278.1221, found: 278.1221.

**5-(Bis(2-cyanoethyl)amino)-[1,2,3]triazolo[4,5-*c*][1,2,5]oxadiazol-5-ium-4-ide (6).** A solution of azidotriazene **7** (870 mg, 3.35 mmol) in MeCN (30 mL) was refluxed for 24 h, and finally allowed to cool to RT. The solvent was evaporated in vacuo and the residue was purified by flash chromatography (ethyl acetate/petroleum ether, 1:1) to give a pale beige solid in a quantitative yield (770 mg). ^1^H NMR (500.1 MHz, [D_6_]-acetone): *δ* 3.22 (t, 4 H, CH_2_C**H**_2_CN, *J* = 6.7 Hz), 4.62 (t, 4 H, C**H**_2_CH_2_CN, *J* = 6.7 Hz) ppm. ^13^C NMR (125.8 MHz, [D_6_]-acetone): *δ* 14.8 (CH_2_**C**H_2_CN), 50.4 (**C**H_2_CH_2_CN), 117.2 (CN), 162.2 (C(3a,6a)) ppm. ^14^N NMR (36.1 MHz, [D_6_]-acetone): *δ* –52 (N-5, ν_½_ = 140 Hz), –130 (N-4, N-6, ν_½_ = 590 Hz) ppm. IR (KBr): *ν*˜ 676, 771, 794, 811, 924, 967, 1014, 1041, 1120, 1192, 1330, 1363, 1392, 1421, 1436, 1480, 1540, 2251, 2938, 2971, 3021 cm^−1^. HRMS (ESI) *m*/*z* [*M*+Na]^+^ calcd for C_8_H_8_N_8_O: 255.0713, found: 255.0705.

**5-((2-Cyanoethyl)amino)-[1,2,3]triazolo[4,5-*c*][1,2,5]oxadiazol-5-ium-4-ide (5).** A solution of KOH (500 mg, 8.88 mmol) in MeOH (15 mL) was added dropwise to a vigorously stirred solution of triazolofurazan **6** (1030 mg, 4.44 mmol) in MeOH (70 mL). The reaction mixture was stirred for 30 min (TLC control), and then the solvent was evaporated in vacuo and the residue was purified by flash chromatography (ethyl acetate) to give a pale yellow solid (742 mg, 93%). ^1^H NMR (600.1 MHz, [D_6_]-acetone): *δ* 3.14 (t, 2 H, CH_2_C**H**_2_CN, *J* = 6.3 Hz), 4.26 (t, 2 H, C**H**_2_CH_2_CN, *J* = 6.3 Hz) ppm. ^13^C NMR (150.9 MHz, [D_6_]-acetone): *δ* 15.7 (CH_2_**C**H_2_CN), 43.6 (**C**H_2_CH_2_CN), 117.2 (CN), 162.4 (C(3a,6a)) ppm. ^14^N NMR (43.4 MHz, [D_6_]-acetone): *δ* –52 (N-5, ν_½_ = 70 Hz), –132 (N-4, N-6, ν_½_ = 620 Hz) ppm. IR (KBr): *ν*˜ 782, 795, 810, 938, 1028, 1051, 1138, 1339, 1360, 1432, 1458, 1548, 2270, 2966, 3015, 3201, 3468 cm^–1^. HRMS (ESI) *m*/*z* [*M*–H]^–^ calcd for C_5_H_5_N_7_O: 178.0483, found: 178.0481.

**5-(*N*-(2-Cyanoethyl)cyanamido)-[1,2,3]triazolo[4,5-*c*][1,2,5]oxadiazol-5-ium-4-ide (4b).** To a stirred solution of triazolofurazan **5** (600 mg, 3.35 mmol) in dry MeCN (5 mL) at RT under an argon atmosphere, a solution of Et_3_N (327 mg, 0.51 mL, 3.69 mmol) in dry MeCN (2 mL) and BrCN (387 mg, 3.69 mmol) in dry MeCN (2 mL) were sequentially added. The reaction mixture was stirred at RT for 30 min (TLC control), then the solvent was evaporated in vacuo and the residue was purified by flash chromatography (ethyl acetate/petroleum ether, 1:1) to give a pale beige solid (563 mg, 82%). ^1^H NMR (500.1 MHz, [D_6_]-acetone): *δ* 3.40 (t, 2 H, CH_2_C**H**_2_CN, *J* = 7.0 Hz), 5.00 (t, 2 H, C**H**_2_CH_2_CN, *J* = 7.0 Hz) ppm. ^13^C NMR (125.8 MHz, [D_6_]-acetone): *δ* 15.5 (CH_2_**C**H_2_CN), 51.1 (**C**H_2_CH_2_CN), 105.1 (CN), 116.0 (CH_2_CH_2_**C**N), 162.2 (C(3a,6a)) ppm. ^14^N NMR (36.1 MHz, [D_6_]-acetone): *δ* –71 (N-5, ν_½_ = 170 Hz), –105 (N-4, N-6, ν_½_ = 500 Hz), –129 (ν_½_ = 480 Hz), –145 (ν_½_ = 600 Hz) ppm. IR (KBr): *ν*˜ 463, 633, 654, 810, 835, 907, 945, 1044, 1213, 1334, 1396, 1434, 1445, 1521, 2248, 2943, 2977, 2990, 3036, 3414 cm^–1^. HRMS (ESI) *m*/*z* [*M*+Na]^+^ calcd for C_6_H_4_N_8_O: 227.0400, found: 227.0406.

**5-((2-Cyanoethyl)(ethoxycarbonyl)amino)-[1,2,3]triazolo[4,5-*c*][1,2,5]oxadiazol-5-ium-4-ide (4c).** To a stirred solution of triazolofurazan **5** (360 mg, 2 mmol) in dry MeCN (5 mL) at RT under an argon atmosphere, a solution of Et_3_N (327 mg, 0.41 mL, 2.6 mmol) in dry MeCN (2.5 mL) and ClCOOEt (284 mg, 0.25 mL, 2.6 mmol) in dry MeCN (2.5 mL) were sequentially added. The reaction mixture was stirred at RT for 24 h (TLC control), then the solvent was evaporated in vacuo and the residue was purified by flash chromatography (ethyl acetate/petroleum ether, 1:1) to give a pale yellow solid (409 mg, 81%). ^1^H NMR (500.1 MHz, [D_6_]-acetone): *δ* 1.31 (t, 3 H, C**H**_3_CH_2_, *J* = 7.0 Hz), 3.08 (t, 2 H, CH_2_C**H**_2_CN, *J* = 6.4 Hz), 4.39 (k, 2 H, CH_3_C**H**_2_, *J* = 7.0 Hz), 4.58 (t, 2 H, C**H**_2_CH_2_CN, *J* = 6.4 Hz) ppm. ^13^C NMR (125.8 MHz, [D_6_]-acetone): *δ* 13.0 (CH_2_**C**H_3_), 16.1 (CH_2_**C**H_2_CN), 47.5 (**C**H_2_CH_2_CN), 64.5 (**C**H_2_CH_3_), 116.5 (CN), 151.6 (C=O), 162.8 (C(3a,6a)) ppm. ^14^N NMR (36.1 MHz, [D_6_]-acetone): *δ* –77 (N-5, ν_½_ = 630 Hz), –130 (500 Hz) ppm. IR (KBr): *ν*˜ 598, 615, 714, 746, 767, 809, 834, 872, 1014, 1029, 1044, 1092, 1153, 1176, 1225, 1291, 1315, 1376, 1402, 1428, 1445, 1466, 1750, 2251, 2981, 3486 cm^−1^.

**K-Salt of 5-(nitroamino)-[1,2,3]triazolo[4,5-*c*][1,2,5]oxadiazol-5-ium-4-ide (3a).** NO_2_BF_4_ (642 mg, 4.8 mmol) was added in one portion to a stirred solution of triazolofurazan **5** (720 mg, 4.0 mmol) in dry MeCN (10 mL) at −30 °C under an argon atmosphere. The reaction mixture was stirred at this temperature for 15 min (TLC control). Then, a cooled (−30 °C) solution of KOH (717 mg, 12.8 mmol) in MeOH (10 mL) was added to the reaction mixture. This was allowed to warm up to RT, then the reaction mixture was concentrated under a reduced pressure. The residue was purified by column chromatography (ethyl acetate/methanol, 5:1) to give a pale yellow solid (737 mg) as a MeCN–nitramide complex (1:1). This complex was dried at 80 °C under reduced pressure for 2 h, yielding K-salt of nitramide (614 mg, 89%) as a yellow solid. ^13^C NMR (125.8 MHz, [D_6_]-DMSO): *δ* 163.0 (C(3a,6a)) ppm. ^14^N NMR (36.1 MHz, [D_6_]-DMSO): *δ* –9 (NO_2_, ν_½_ = 40 Hz), –48 (N-5, ν_½_ = 180 Hz). ^13^C NMR of MeCN–nitramide complex (1:1) (150.9 MHz, [D_6_]-acetone): *δ* 163.2 (C(3a,6a)) ppm. ^14^N NMR of MeCN–nitramide complex (1:1) (43.4 MHz, [D_6_]-acetone): *δ* 23 (N-1 and N-3, ν_½_ = 555 Hz), –8 (NO_2_, ν_½_ = 40 Hz), –50 (N-5, ν_½_ = 200 Hz), –89 (N-4 and N-6 or **N**-NO_2_, ν_½_ = 440 Hz) ppm. ^15^N NMR of MeCN–nitramide complex (1:1) (60.8 MHz, [D_6_]-acetone): *δ* 23 (N-1 and N-3), –8 (NO_2_), –49 (N-5), –87, 94 (N-4, N-6, **N**-NO_2_) ppm. IR (KBr): *ν*˜ 656, 751, 829, 1029, 1054, 1178, 1241, 1311, 1446 cm^−1^. Elemental analysis calculated (%) for C_2_KN_7_O_2_: C 11.48, H 0.00, N 46.88; found C 11.59, H 0.00, N 46.61.

**Na-Salt of 5-cyanamido-[1,2,3]triazolo[4,5-*c*][1,2,5]oxadiazol-5-ium-4-ide dihydrate (3b).** To a stirred solution of triazolofurazan **4b** (100 mg, 0.5 mmol) in dry THF (5 mL) at RT under an argon atmosphere, a 2M THF solution of NaHMDS (alternatively, 2 eq. of DBU with subsequent treatment with ion-exchange resins (Amberlite IR 120, Na-form) can be used) (0.25 mL, 0.5 mmol) was added. The reaction mixture was stirred at RT for 5 min (TLC control), then the reaction mixture was purified by flash chromatography (ethyl acetate/methanol, 5:1) to give Na-salt dihydrate as a yellow solid (87 mg, 85%). ^13^C NMR (150.9 MHz, [D_4_]-methanol): *δ* 117.6 (CN), 163.5 (C(3a,6a)) ppm. ^14^N NMR (43.4 MHz, [D_4_]-methanol): *δ* –31 (N-5, ν_½_ = 60 Hz), –125 (ν_½_ = 800 Hz), –176 (ν_½_ = 505 Hz) ppm. IR (KBr): *ν*˜ 532, 551, 805, 825, 883, 940, 1042, 1158, 1352, 1386, 1440, 1526, 1637, 2171, 2191, 2280, 2928, 3508 cm^−1^. Elemental analysis calcd (%) for C_3_H_4_N_7_NaO_3_: C 17.23, H 1.93, N 46.89; found C 17.11, H 2.01, N 46.45.

**Na-Salt of [1,2,3]triazolo[4,5-*c*][1,2,5]oxadiazol-5-ium-4-id-5-yl(ethoxycarbonyl)amide monohydrate (3c).** To a stirred solution of triazolofurazan **4c** (190 mg, 0.757 mmol) in dry THF (5 mL) at RT under an argon atmosphere, a 2M THF solution of NaHMDS (0.38 mL, 0.76 mmol) was added. The reaction mixture was stirred at RT for 5 min (TLC control), then the reaction mixture was purified by flash chromatography (ethyl acetate/methanol, 5:1) to give Na-salt monohydrate as a deep yellow solid (162 mg, 90%). ^1^H NMR (600.1 MHz, [D_6_]-DMSO): *δ* 1.19 (t, 3 H, C**H**_3_CH_2_, *J* = 6.6 Hz), 4.03 (k, 2 H, CH_3_C**H**_2_, *J* = 6.6 Hz) ppm. ^13^C NMR (150.9 MHz, [D_6_]-DMSO): *δ* 14.6 (CH_2_**C**H_3_), 59.5 (**C**H_2_CH_3_), 158.9 (C=O), 162.6 (C(3a,6a)) ppm. ^14^N NMR (43.4 MHz, [D_6_]-DMSO): *δ* –29 (N-5, ν_½_ = 60 Hz) ppm. IR (KBr): *ν*˜ 510, 779, 819, 1094, 1147, 1288, 1360, 1380, 1450, 1639, 3176, 3269, 3437, 3635 cm^–1^. Elemental analysis calcd (%) for C_5_H_7_N_6_NaO_4_: C 25.22, H 2.96, N 35.29; found C 25.39, H 3.05, N 34.72.

**X-ray crystallographic data and refinement details**.

X-ray diffraction data for **3a**, **3b,** and **3b** were collected on a Bruker Smart APEX II diffractometer equipped with a Photon II detector, for **4b** on a Bruker Quest diffractometer equipped with a Photon III detector, and for **6** and **7** on a Bruker APEX DUO diffractometer equipped with a CCD area detector. For all of the experiments, MoKα radiation was used (λ = 0.71072 Å, graphite monochromator). A semiempirical absorption correction was applied with the SADABS program [26] using the intensity data of equivalent reflections. Structures were solved with the dual-space method using the SHELXT program [27] and refined by a full-matrix least squares technique on *F*^2^ with anisotropic displacement parameters for non-hydrogen atoms with the SHELXL program [28]. Hydrogen atoms of the coordinated water molecules in **3b** and **3c** were found from difference Fourier synthesis and were refined in isotropic approximation. All of the other hydrogen atoms were placed in calculated positions and refined in the riding model with isotropic displacement parameters *U*_iso_(H) equal to 1.5*U*_eq_(C) for methyl groups and 1.2*U*_eq_(C) for the other groups. Detailed crystallographic information is provided in the Supplementary Materials. Full crystallographic data have been deposited with the Cambridge Crystallographic Data Center, CCDC 2183138–2183143. Copies of the data can be obtained free of charge via https://www.ccdc.cam.ac.uk/structures/ (accesses on 24 August 2022).

## Figures and Tables

**Table 1:** Selected bond lengths of compounds **3a**–**c**, **4b**, and **6**.

Compound	3a	3b	3c	4b	6	12 ^1^
bond lengths, Å						
N4–N5	1.337(2)	1.3420(18)	1.360(2)	1.328(2)	1.3465(8)	1.3545(16)
N5–N6	1.339(2)	1.3535(17)	1.347(2)	1.324(2)	1.3465(8)	1.3443(16)
N5–N7	1.382(2)	1.3368(18)	1.338(2)	1.379(2)	1.3320(14)	-

^1^ Lit. [10].

## Data Availability

Data obtained in this project are contained within this article and are available upon request from the authors.

## Supplementary Materials

The following supporting information can be downloaded at: https://www.mdpi.com/article/10.3390/molecules27196287/s1. Crystallographic data (Figures S1–S10, Tables S1 and S2). Thermal behavior (Figures S11–S19, Table S3). NMR and IR spectra.

## Sample Availability

Samples of the compounds are not available from the authors.
