# Supplementary material for: Anions Containing Tripoid Conjugated N4− System: Salts of 5-(Substituted Amino)-[1,2,3]triazolo[4,5-c][1,2,5]oxadiazol-5-ium-4-ides, as well as Their Synthesis, Structure, and Thermal Stability
Source: Molecules. 2022 Sep 23;27(19):6287. doi: 10.3390/molecules27196287 (PMC9571959; doi:10.3390/molecules27196287)
Supplement: Supplementary file 1 [file molecules-27-06287-s001.zip › molecules-1905183-supplementary.pdf]

Supporting Information

Anions Containing Tripoid Conjugated N_4^- System: Salts of 5-(Substituted Amino)-[1,2,3]triazolo[4,5-*c*][1,2,5]oxadiazol-5-ium-4-ides, as well as their Synthesis, Structure, and Thermal Stability

Alexey A. Voronin [1,*], Sofya P. Balabanova [1], Ivan V. Fedyanin [2], Aleksandr M. Churakov [1,*], Alla N. Pivkina [3], Yurii A. Strelenko [1], Michael S. Klenov [1] and Vladimir A. Tartakovsky [1]

[1] N. D. Zelinsky Institute of Organic Chemistry, Russian Academy of Sciences, 47 Leninsky prosp., 119991 Moscow, Russia

[2] A. N. Nesmeyanov Institute of Organoelement Compounds, Russian Academy of Sciences, 28 Vavilova St., 119991 Moscow, Russa

[3] N. N. Semenov Federal Research Center for Chemical Physics, Russian Academy of Sciences, 4 Kosygina St., Building 1, 119991 Moscow, Russia

Xray crystallographic data

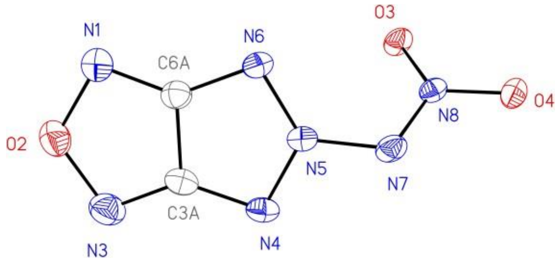

Figure S1. General view of the anion **3a** in crystal. Anisotropic displacement parameters are drawn at 50% probability level.

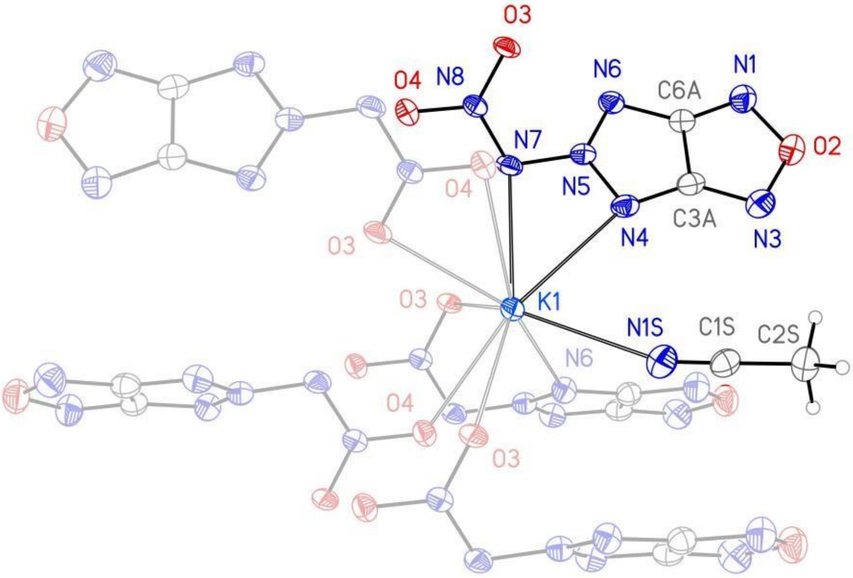

Figure S2.The environment of the potassium cation in crystal **3a** demonstrating its coordination by different heteroatoms of the anion and the acetonitrile molecule. The moieties constituting the asymmetric part of the unit cell are highlighted.

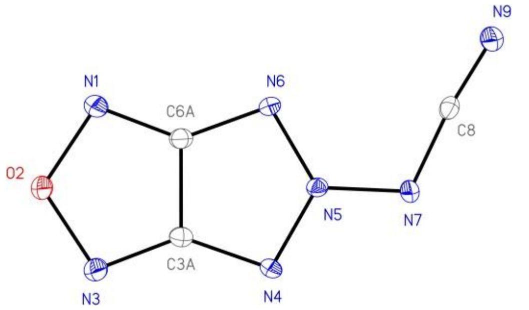

Figure S3. General view of the anion **3b** in crystal. Anisotropic displacement parameters are drawn at 50% probability level.

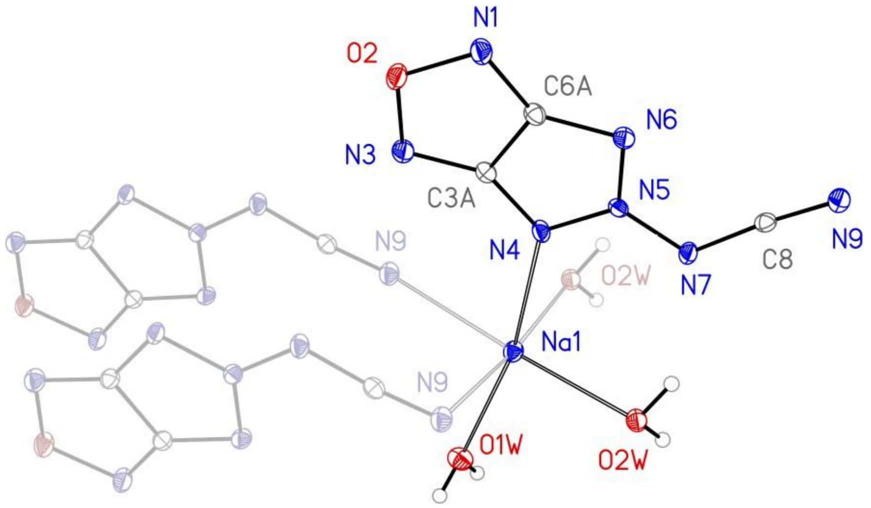

Figure S4.The environment of the sodium cation in crystal **3b** demonstrating its coordination by different heteroatoms of the anion and water molecules. The moieties constituting the asymmetric part of the unit cell are highlighted.

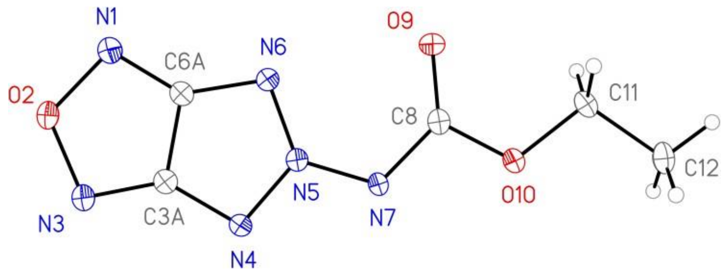

Figure S5. General view of the anion **3c** in crystal. Anisotropic displacement parameters are drawn at 50% probability level.

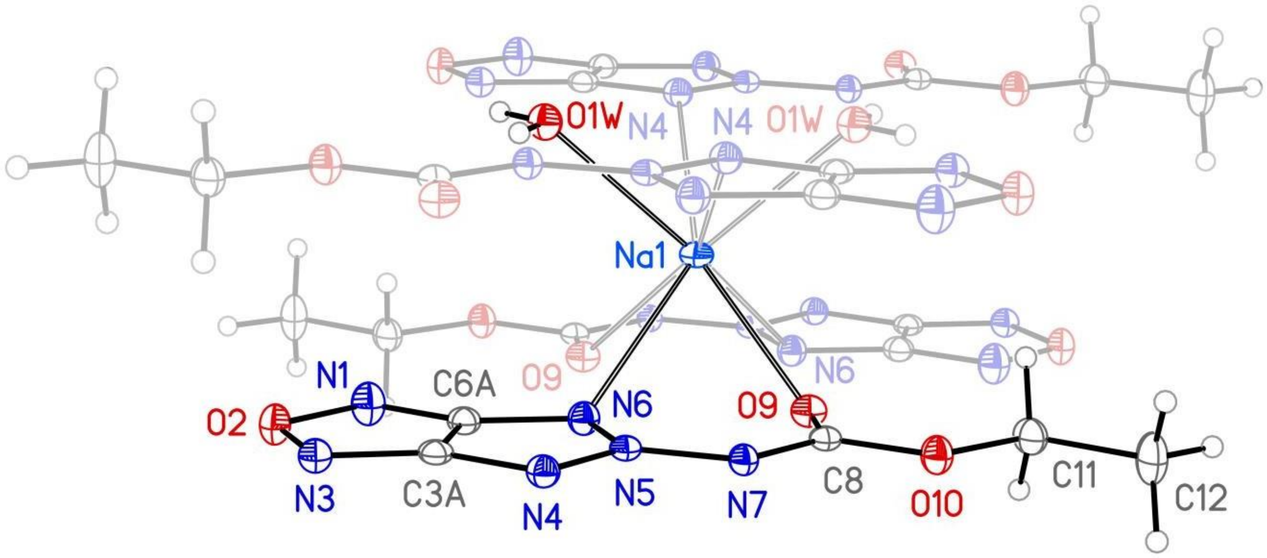

Figure S6.The environment of the sodium cation in crystal **3b** demonstrating its coordination by different heteroatoms of the anion and water molecules. The moieties constituting the asymmetric part of the unit cell are highlighted. The anion and the water molecule lie on a mirror plane and the sodium cation lies on the intersection of a 2-fold axis and a glide plane (sp. gr. *C*2/*m*).

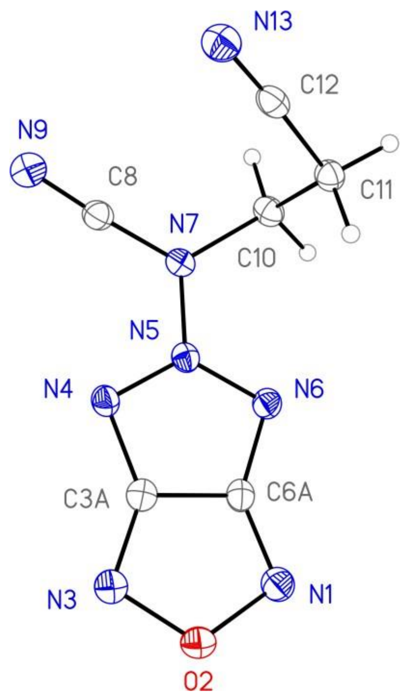

Figure S7. General view of the molecule **4b** in crystal. Anisotropic displacement parameters are drawn at 50% probability level.

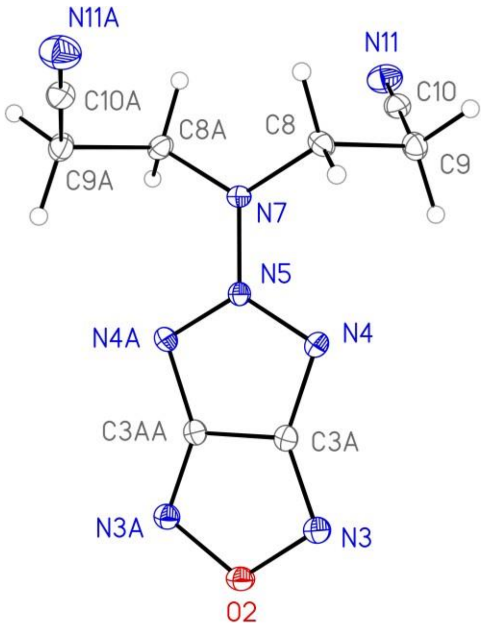

Figure S8. General view of the molecule **6** in crystal. Anisotropic displacement parameters are drawn at 50% probability level.

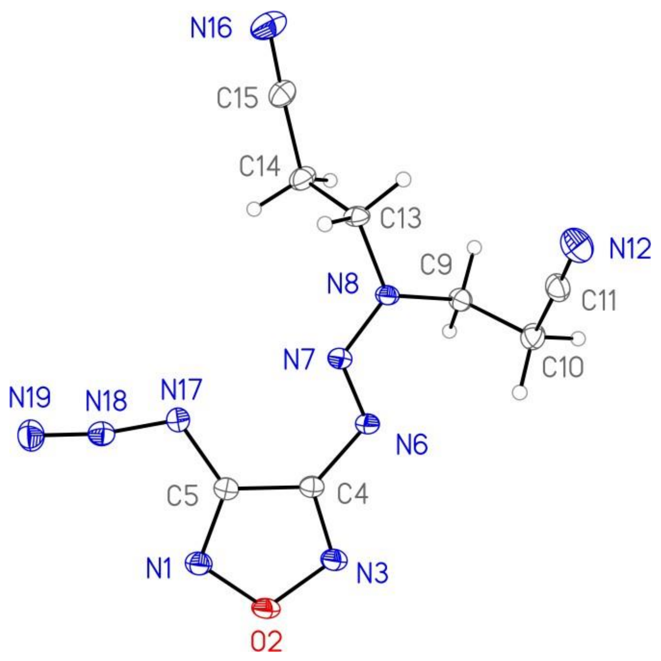

Figure S9. General view of the molecule **7** in crystal. Anisotropic displacement parameters are drawn at 50% probability level.

Table S1. Crystallographic data for crystals of compounds **3a–c**, **4b**, **6–7**.

compound	**3a**	**3b**	**3c**	**4b**	**6**	**7**
CCDC	2183138	2183139	2183140	2183141	2183142	2183143
empirical formula	$C_4H_3KN_8O_3$	$C_3H_4N_7NaO_3$	$C_5H_7N_6NaO_4$	$C_6H_4N_8O$	$C_8H_8N_8O$	$C_8H_8N_{10}O$
T, K	250.24	209.12	238.16	204.17	232.22	260.24
crystal system	140	100	100	120	100	120
space group	$P2_1/c$	$Pna2_1$	$C2/m$	$P2_1$	$C2/c$	$C2/c$
Z / Z'	4 / 1	4 / 1	4 / 0.5	2 / 1	4 / 0.5	8 / 1
a, Å	16.0696(12)	12.2360(3)	13.1460(11)	6.5170(4)	13.0457(4)	20.5474(19)
b, Å	5.7168(4)	17.6766(3)	6.2603(5)	5.4602(4)	11.8643(4)	6.4371(6)
c, Å	10.2887(7)	3.58020(10)	12.4249(11)	12.1137(8)	6.5869(2)	17.5774(17)
β, °	91.457(3)		117.552(2)	105.382(3)	100.2912(18)	93.468(2)
V, Å^3	944.88(12)	774.36(3)	906.58(13)	415.61(5)	1003.11(6)	2320.6(4)
d_{calc}, g cm^{-3}	1.759	1.794	1.745	1.631	1.538	1.490
μ(MuKα), mm^{-1}	5.72	2.00	1.88	1.25	1.14	1.12
$2\theta_{max}$, °	60.0	60.0	60.0	66.3	60.0	60.0
collected reflns.	11049	19383	7565	6470	5948	9893
unique reflns.	2727	2235	1438	3068	1473	3357
reflns. with $I > 2\sigma(I)$	2347	2128	1266	2561	1336	2787
R_1	0.0427	0.0274	0.0438	0.0375	0.0330	0.0356
wR_2	0.1134	0.0713	0.1101	0.0827	0.0910	0.0940
GOF on F^2	1.066	1.049	1.058	1.010	1.038	1.044
ρ_{max} / ρ_{min}, e Å^{-3}	0.743/-0.363	0.452/-0.211	0.724/-0.527	0.310/-0.197	0.499/-0.295	0.376/-0.227

Table S2. Selected bond lengths and angles of the heterocycles in compounds **3a-c**, **4b**, **6-7**.

compound	**3a**	**3b**	**3c**	**4b**	**6***	**7**
	bond lengths, Å					
N1–O2	1.407(2)	1.4056(18)	1.405(2)	1.399(2)	1.4118(8)	1.3916(11)
O2–N3	1.400(3)	1.4087(18)	1.408(2)	1.402(2)	1.4118(8)	1.3854(11)
N1–C6A	1.315(3)	1.308(2)	1.311(3)	1.314(3)	1.3114(11)	1.3027(13)
N3–C3A	1.319(3)	1.310(2)	1.310(3)	1.318(3)	1.3114(11)	1.3097(12)
N4–C3A	1.358(3)	1.3695(19)	1.363(3)	1.371(3)	1.3693(10)	
N6–C6A	1.371(3)	1.361(2)	1.368(3)	1.376(2)	1.3693(10)	
N4–N5	1.337(2)	1.3420(18)	1.360(2)	1.328(2)	1.3465(8)	
N5–N6	1.339(2)	1.3535(17)	1.347(2)	1.324(2)	1.3465(8)	
C3A–C6A	1.408(3)	1.414(2)	1.406(3)	1.406(3)	1.4177(15)	1.4381(14)
N5–N7	1.382(2)	1.3368(18)	1.338(2)	1.379(2)	1.3320(14)	
	bond angles, °					
N1–O2–N3	113.97(15)	113.11(11)	113.27(15)	114.05(13)	113.47(8)	111.05(7)
O2–N1–C6A	101.59(17)	102.58(12)	102.47(17)	102.13(16)	102.19(6)	105.12(8)
O2–N3–C3A	102.35(17)	102.49(12)	102.03(16)	101.79(16)	102.19(6)	105.79(8)
N1–C6A–3A	111.71(18)	110.99(14)	110.77(18)	110.96(19)	111.08(5)	108.36(8)
N3–C3A–C6A	110.39(19)	110.82(13)	111.46(19)	111.08(19)	111.08(5)	109.67(9)
C3A–C6A–N6	109.48(17)	109.87(13)	109.70(18)	109.52(18)	109.84(4)	
C6A–C3A–N4	110.11(17)	109.47(14)	110.02(18)	109.73(18)	109.84(4)	
C6A–N6–N5	99.44(15)	100.29(12)	100.73(16)	98.93(16)	99.53(7)	
C3A–N4–N5	99.70(16)	100.44(12)	100.46(16)	98.94(16)	99.53(7)	
N4–N5–N6	121.25(16)	119.92(13)	119.09(16)	122.87(14)	121.27(9)	
N4–N5–N7	114.84(15)	117.27(12)	114.18(16)	118.60(16)	119.37(5)	
N6–N5–N7	123.39(16)	122.81(13)	126.73(17)	118.50(16)	119.37(5)	

*Some bond lengths and angles for **6** are pairwise identical due to C_2 molecular symmetry in the crystal.

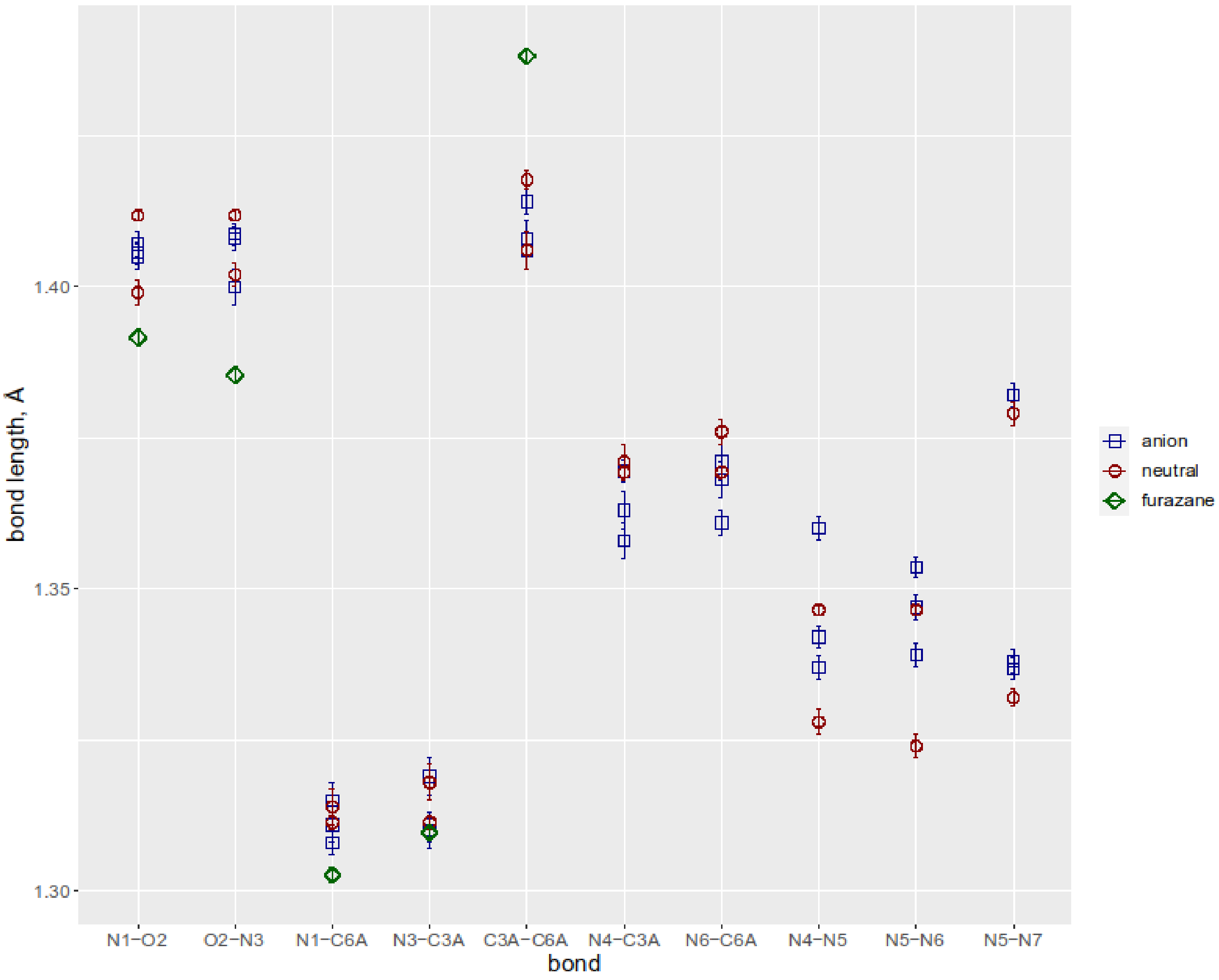

Figure S10. Graphical representation of bond length distribution in anions (**3a-3c)**, neutral molecules (**4b** and **6**) and substituted furazane **7**. The s.u. values from the least square refinement are provided with error bars.

Thermal behavior

Table S3. Summary of thermal stability data

Compound	Melting point, °C	Decomposition onset,* °C
**3a**	152	152
**3b**	–	190
**3c**	–	122
**4b**	76	185
**4c**	61	233
**5**	127	127
**6**	139	234
**7**	79	79 (221)

*The extrapolated onset temperature of the exothermic peak by DSC at 5 K min^{-1} rate.

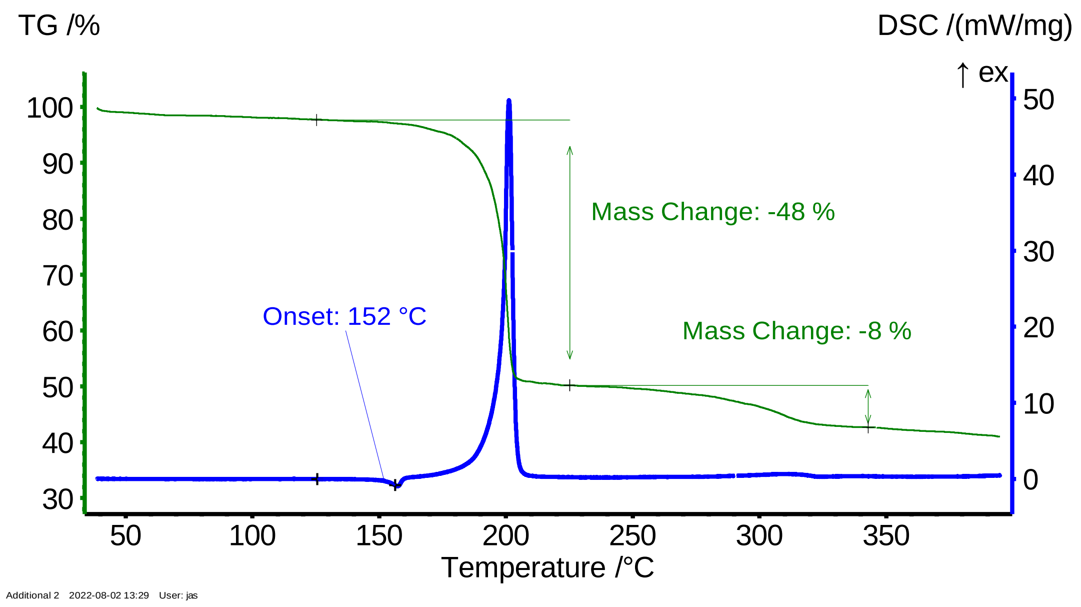

Figure S11. DSC (blue curve) and mass loss (TG, green curve) signals for **3a** heated at 5 K min^{-1} rate.

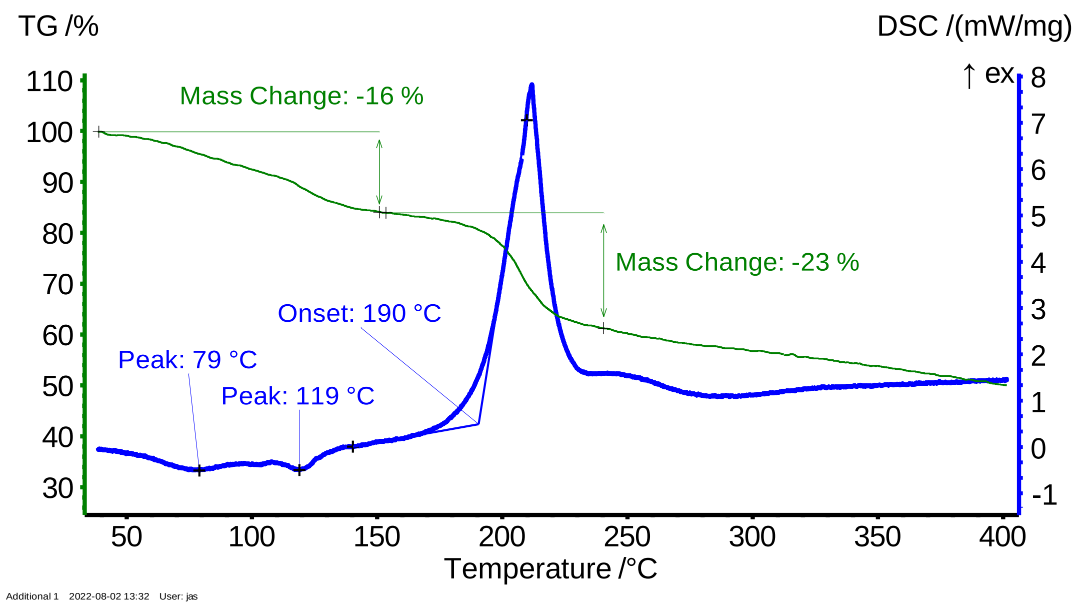

Figure S12. DSC (blue curve) and mass loss (TG, green curve) signals for **3b** heated at 5 K min^{-1} rate.

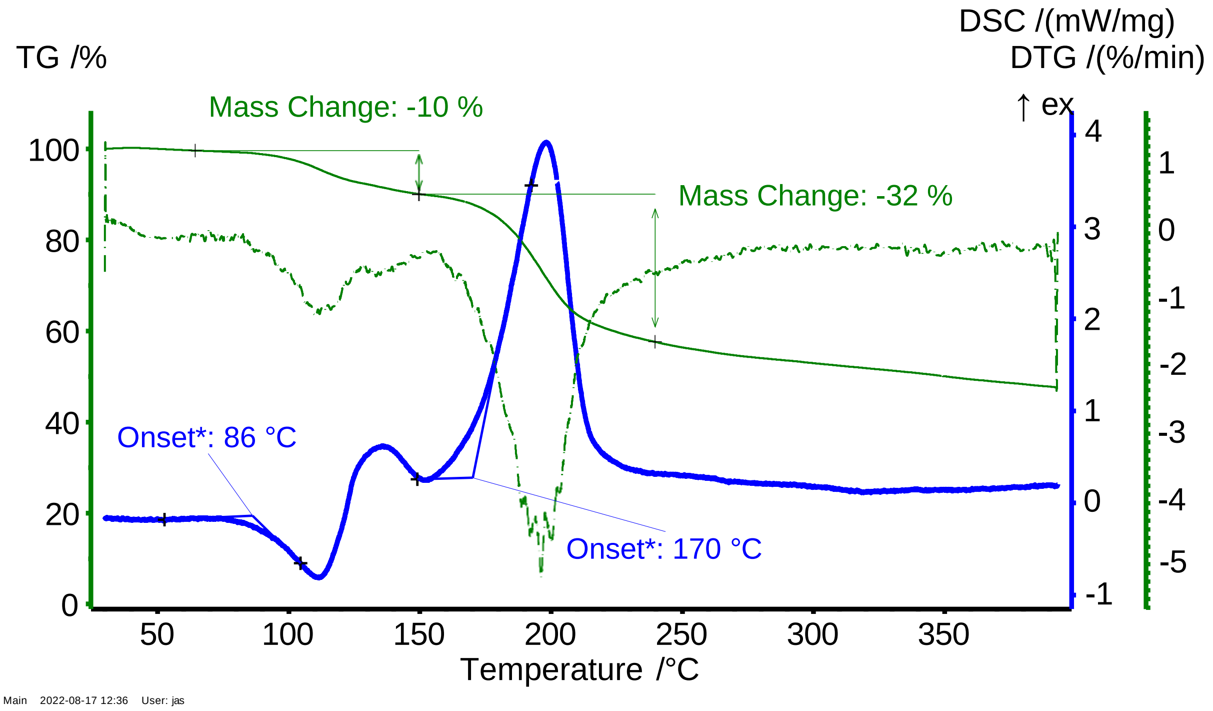

Figure S13. DSC/TG signal for **3c** heated at 5 K min⁻¹ rate.

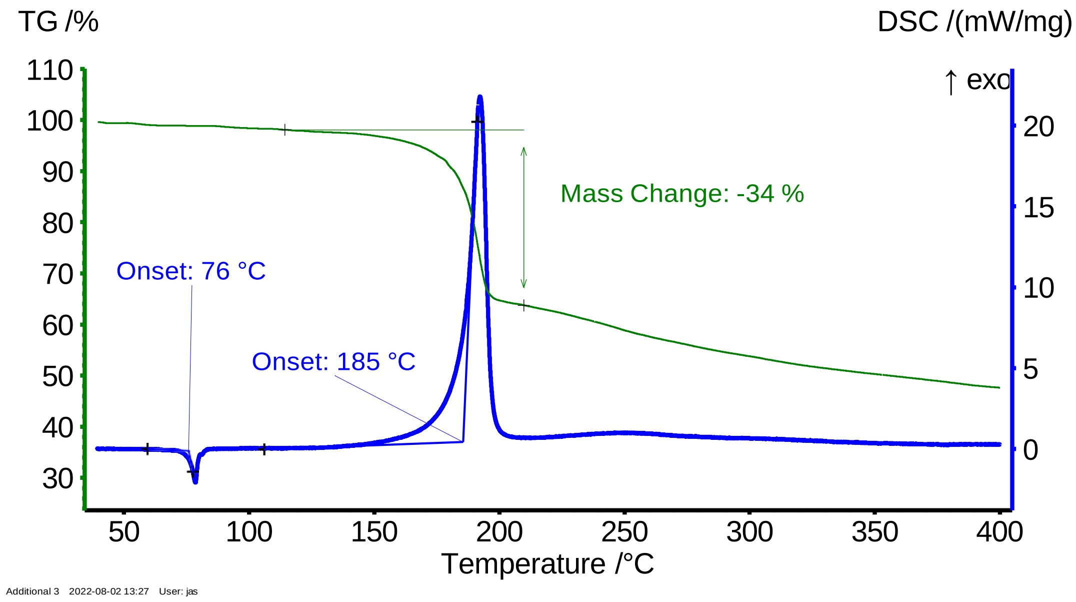

Figure S14. DSC (blue curve) and mass loss (TG, green curve) signals for **4b** heated at 5 K min^{-1} rate.

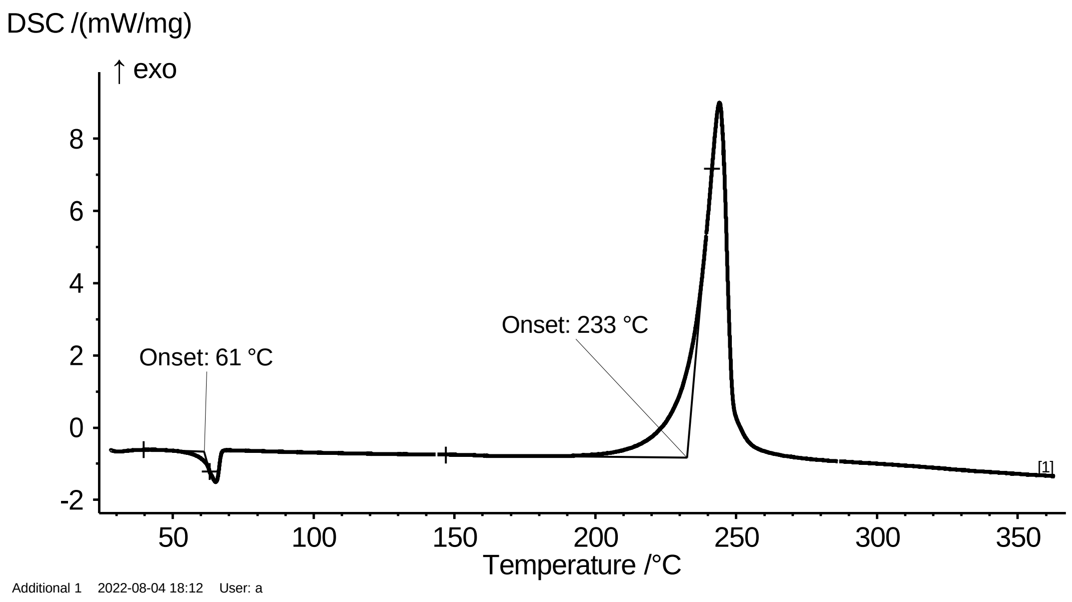

Figure S15. DSC signal for **4c** heated at 5 K min⁻¹ rate.

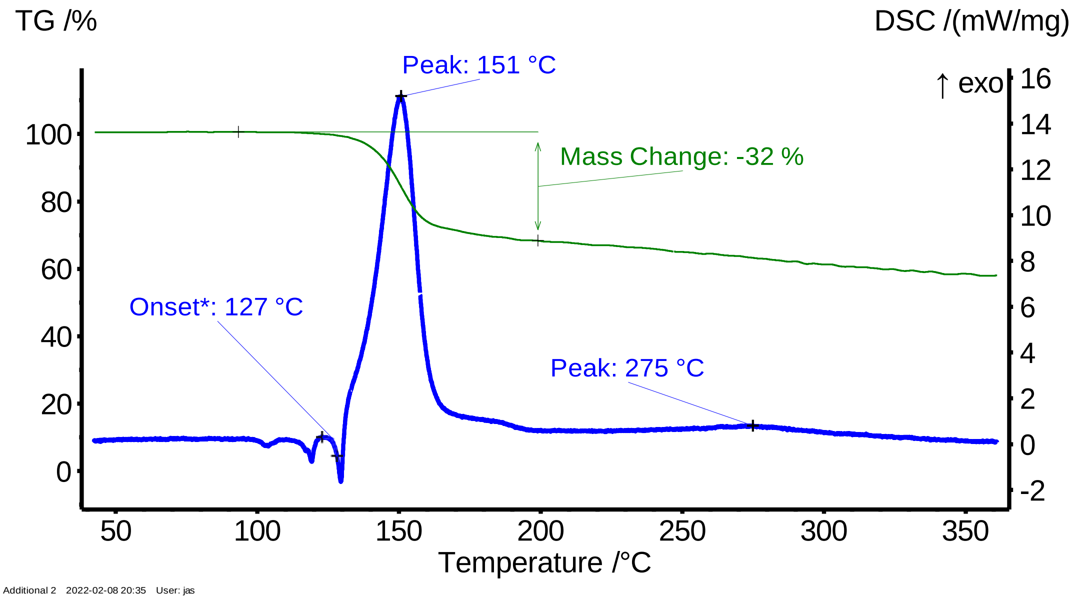

Figure S16. DSC (blue curve) and mass loss (TG, green curve) signals for **5** heated at 5 K min⁻¹ rate.

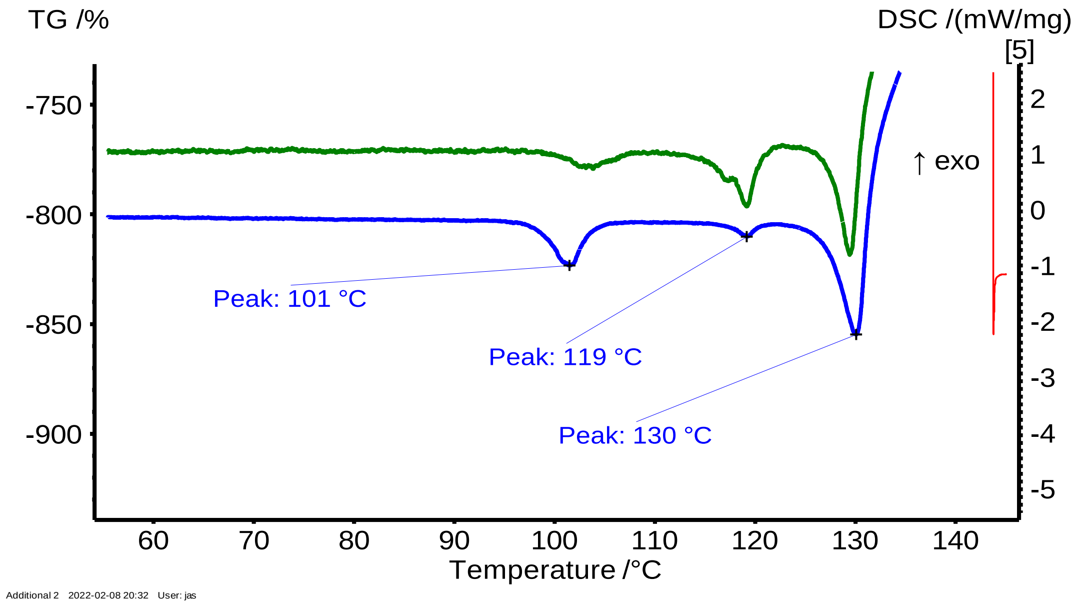

Figure S17. The magnified view of DSC (blue curve) signals for **5** heated at 5 K min^{-1} rate: upper curve corresponds to an experiment with 0.46 mg sample mass, bottom – with 1.56 mg of the sample.

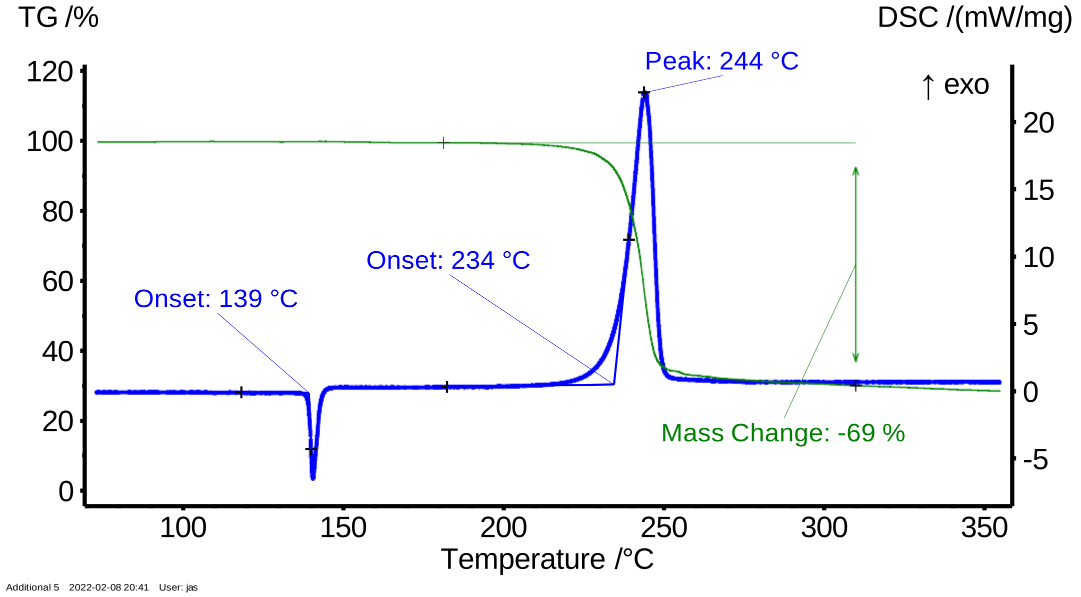

Figure S18. DSC (blue curve) and mass loss (TG, green curve) signals for **6** heated at 5 K min^{-1} rate.

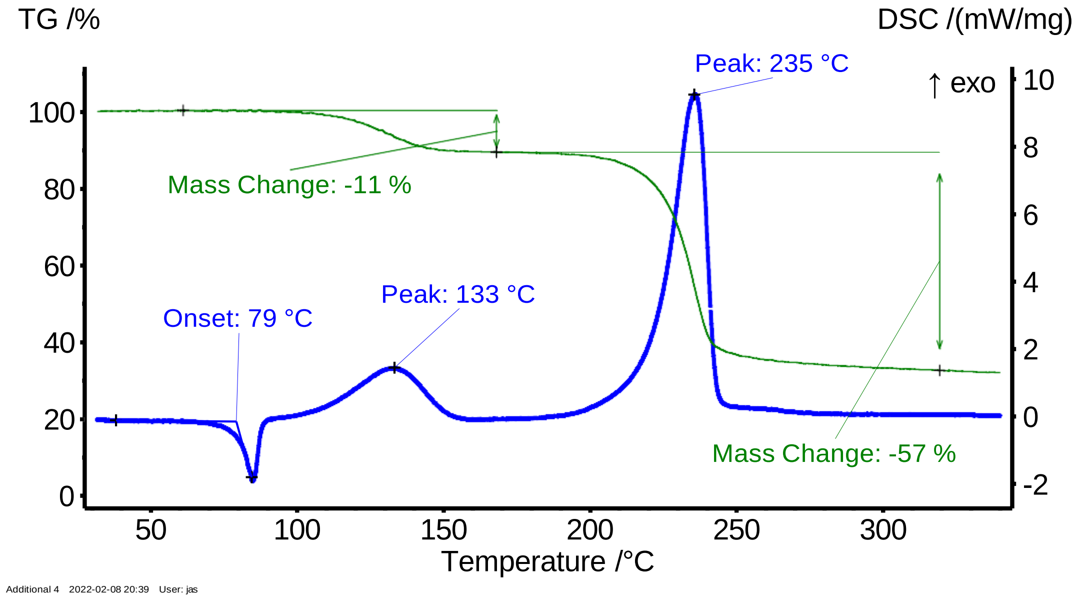

Figure S19. DSC (blue curve) and mass loss (TG, green curve) signals for **7** heated at 5 K min^{-1} rate.

NMR and IR spectra for compound 7

H NMR (500.1 MHz, acetone-d6)

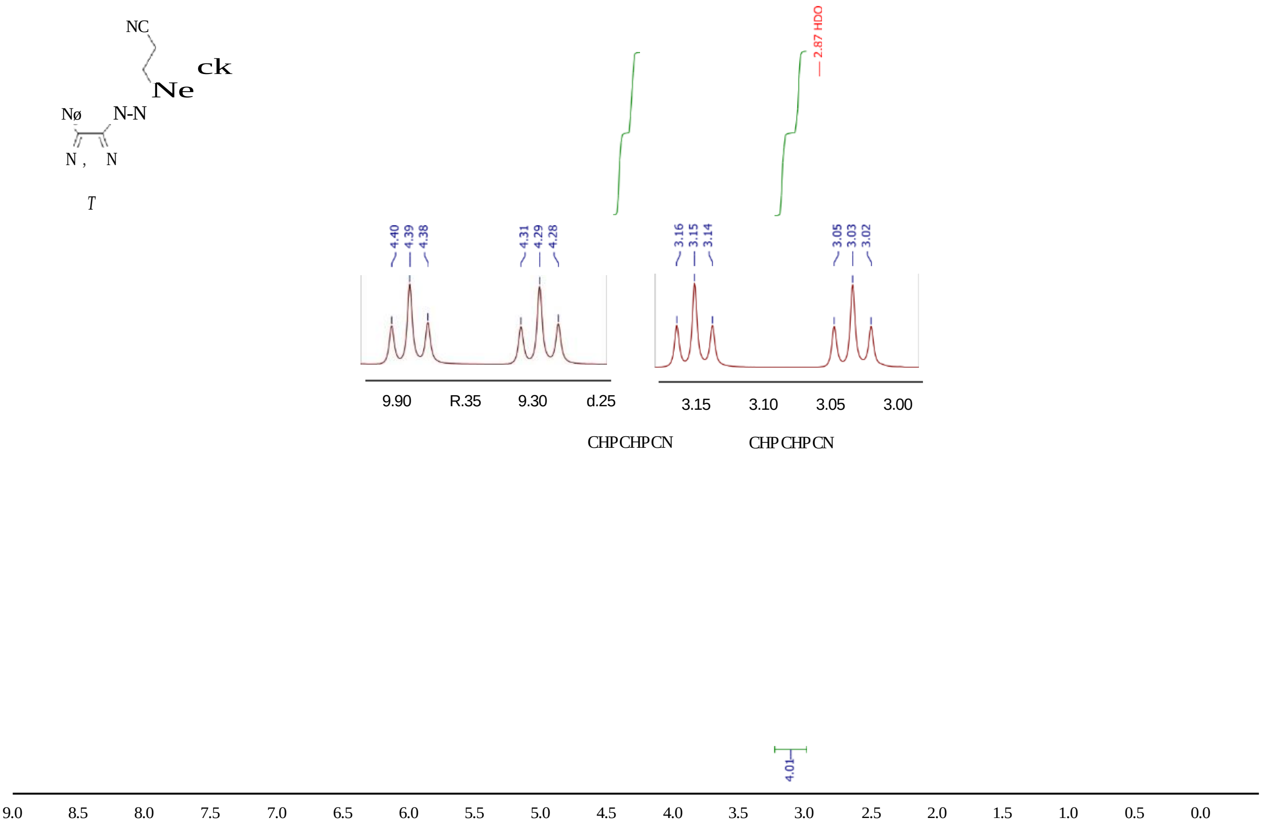

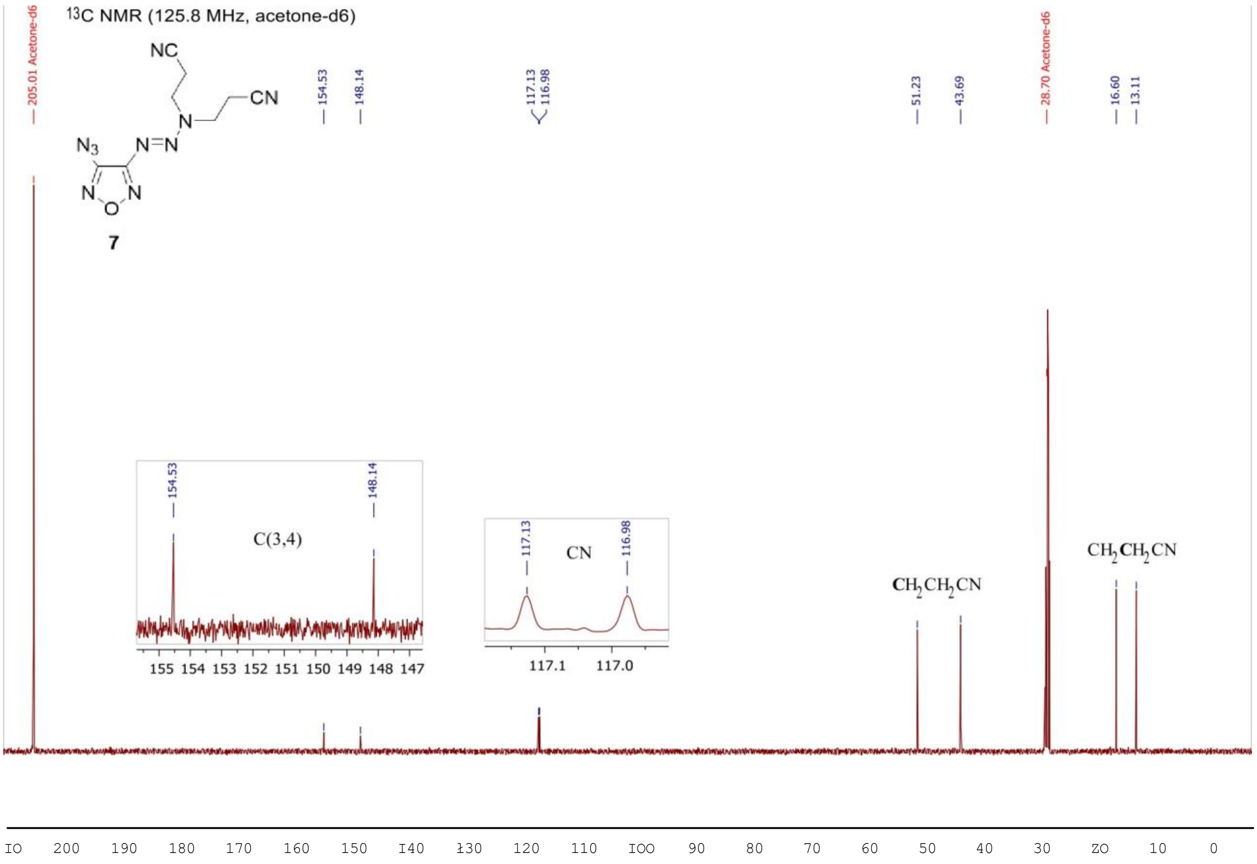

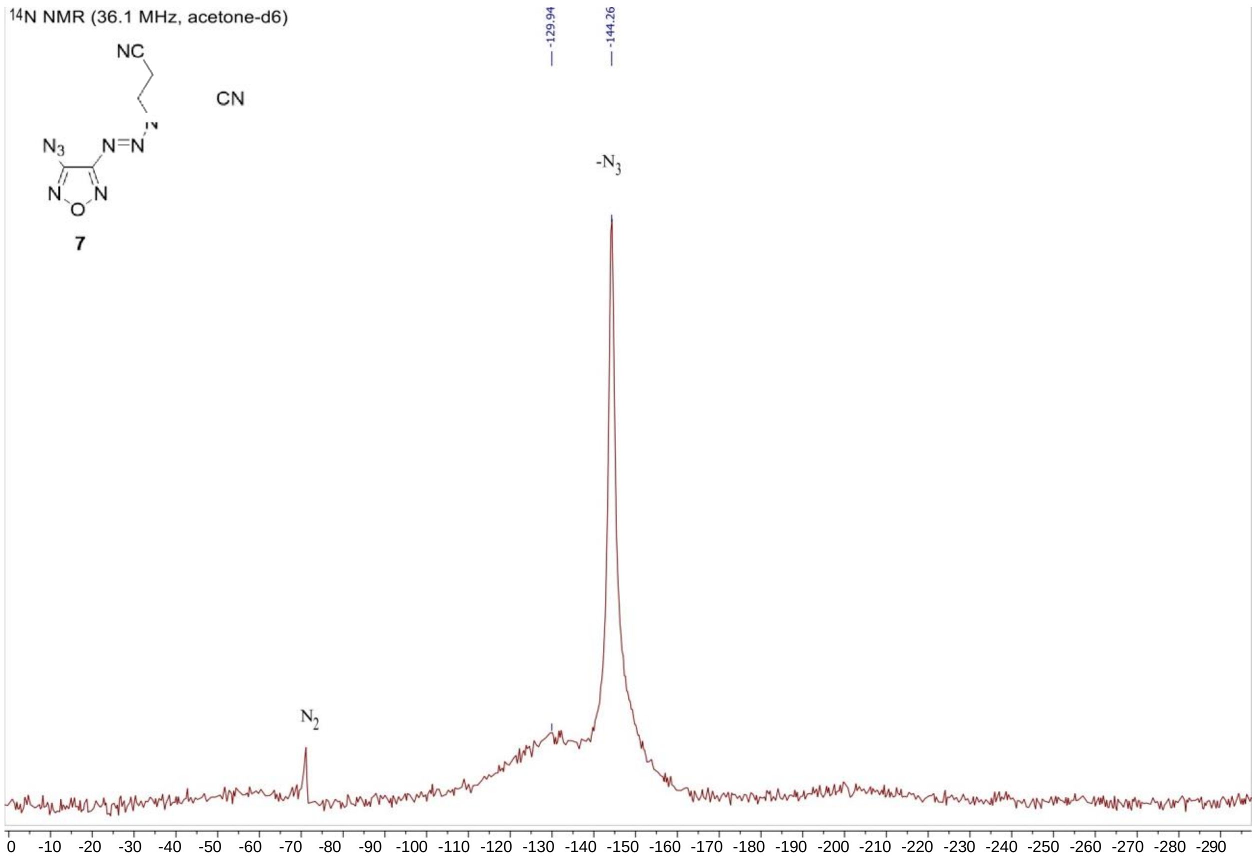
14N NMR (36.1 MHz, acetone-d6)
-129.94
-144.26
NC
CN
N3
N=N
N
N
O
7
-N3
N2
0 -10 -20 -30 -40 -50 -60 -70 -80 -90 -100 -110 -120 -130 -140 -150 -160 -170 -180 -190 -200 -210 -220 -230 -240 -250 -260 -270 -280 -290

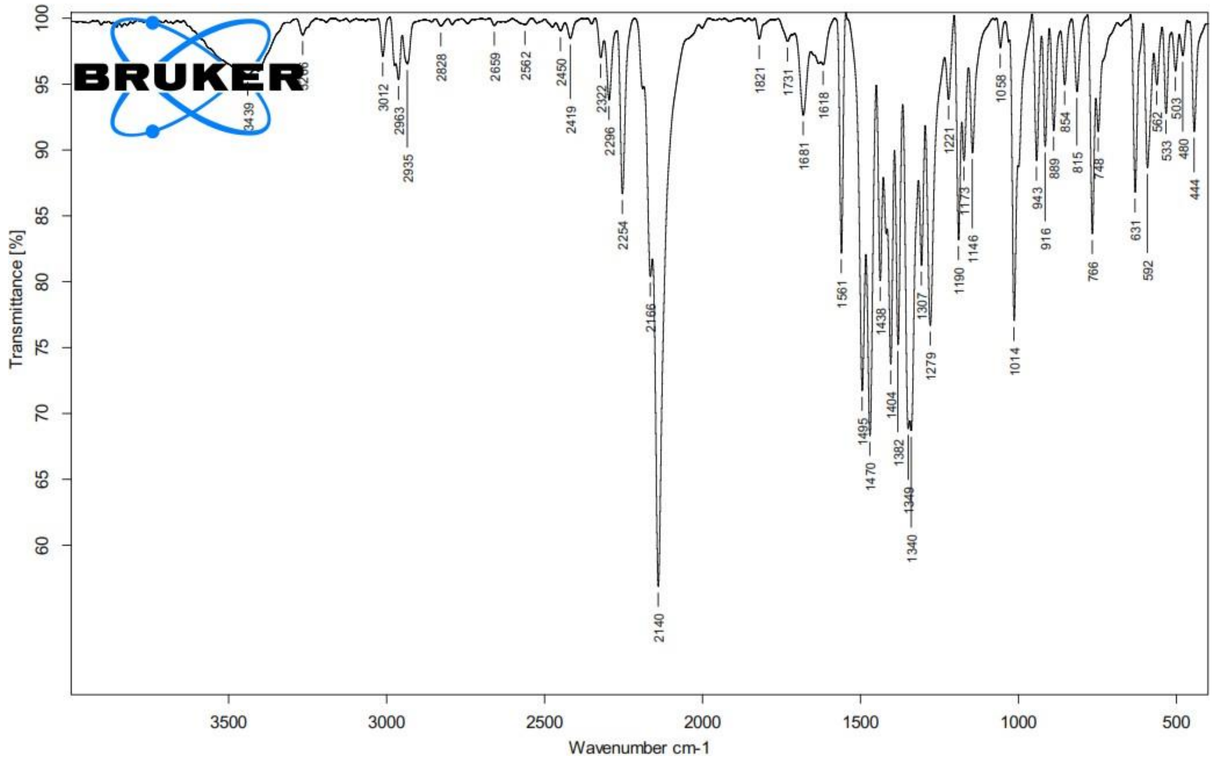
BRUKER
Transmittance [%]
Wavenumber cm-1
3500 3000 2500 2000 1500 1000 500
100 95 90 85 80 75 70 65 60
3439
3012
2963
2935
2828
2659
2562
2450
2419
2322
2296
2254
2166
2140
1821
1731
1681
1618
1561
1495
1470
1438
1404
1382
1349
1340
1307
1279
1221
1190
1173
1146
1058
1014
943
916
889
854
815
766
748
631
592
562
533
503
480
444

NMR and IR spectra for compound 6

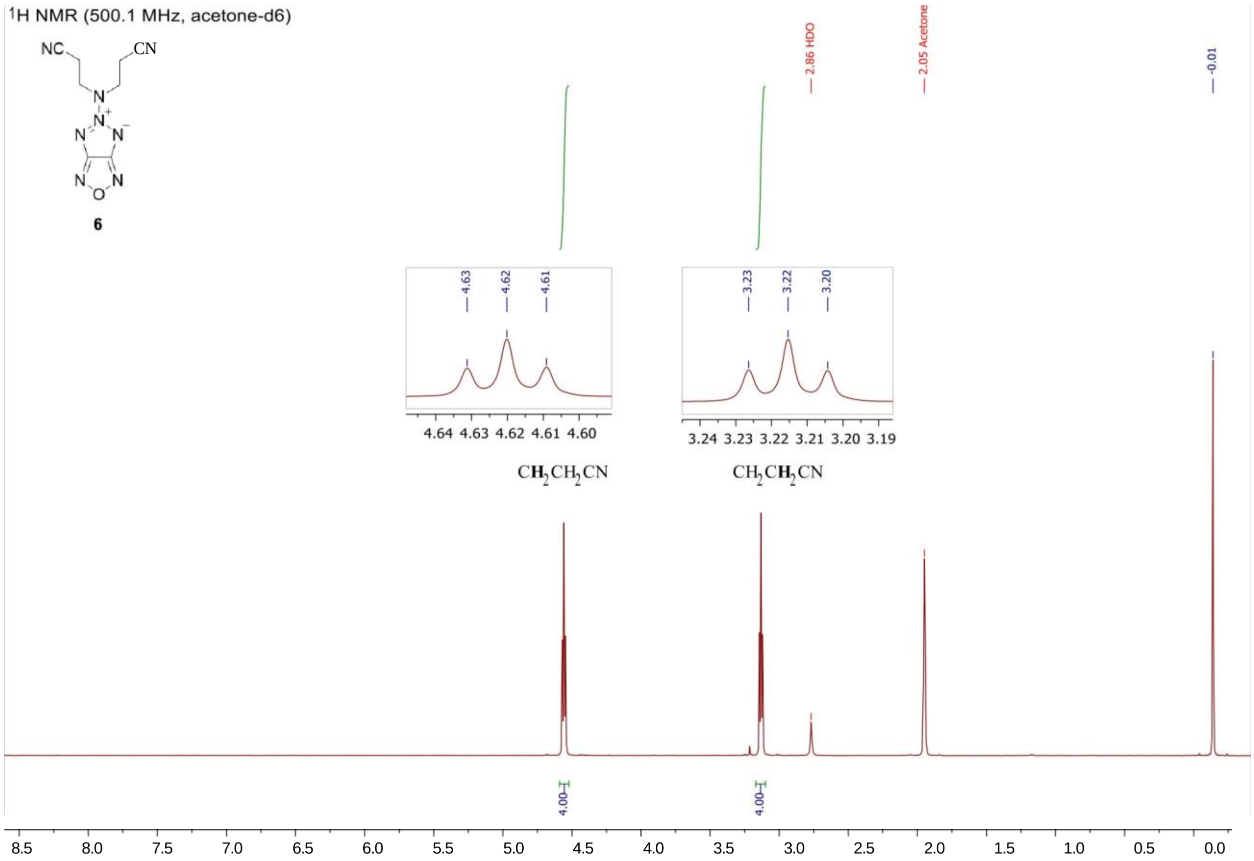

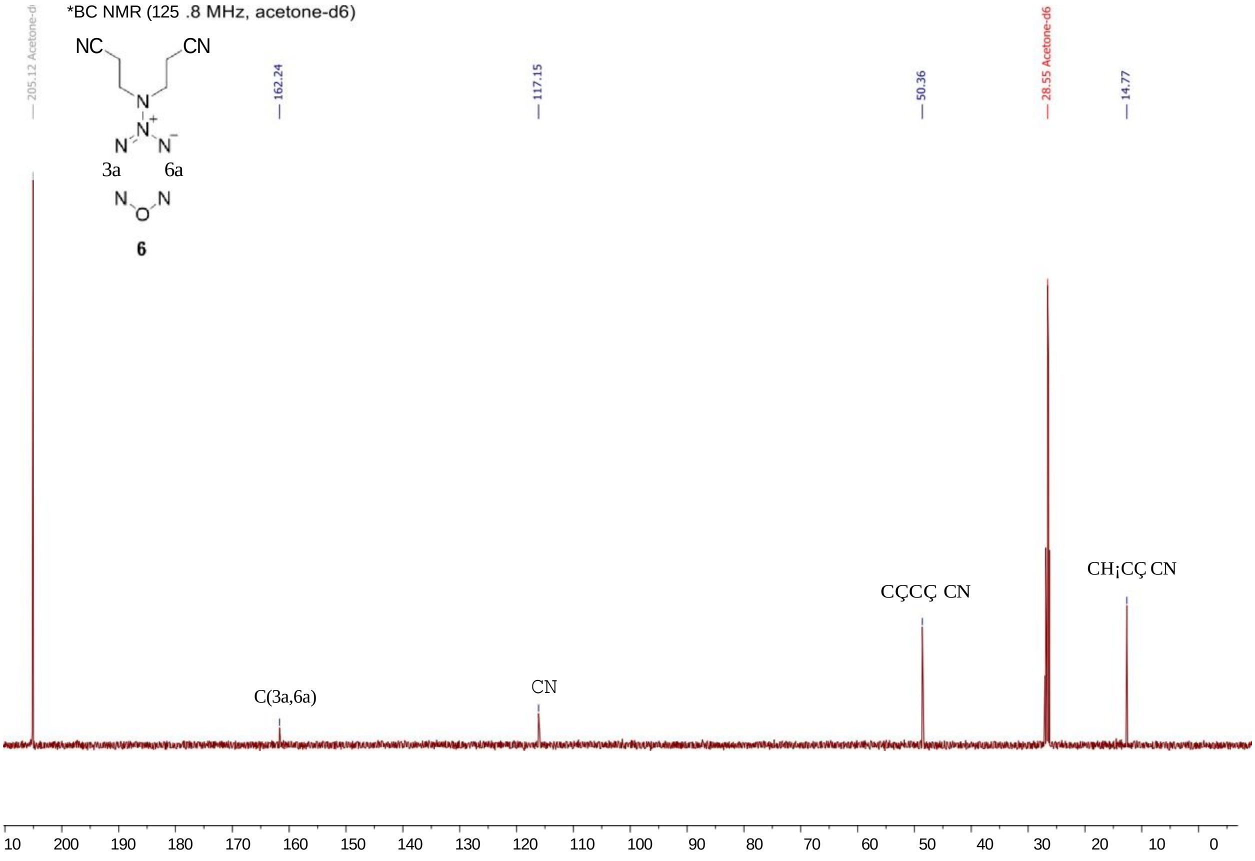

^{14}N NMR (36.1 MHz, acetone-d6)

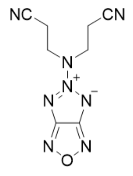

6

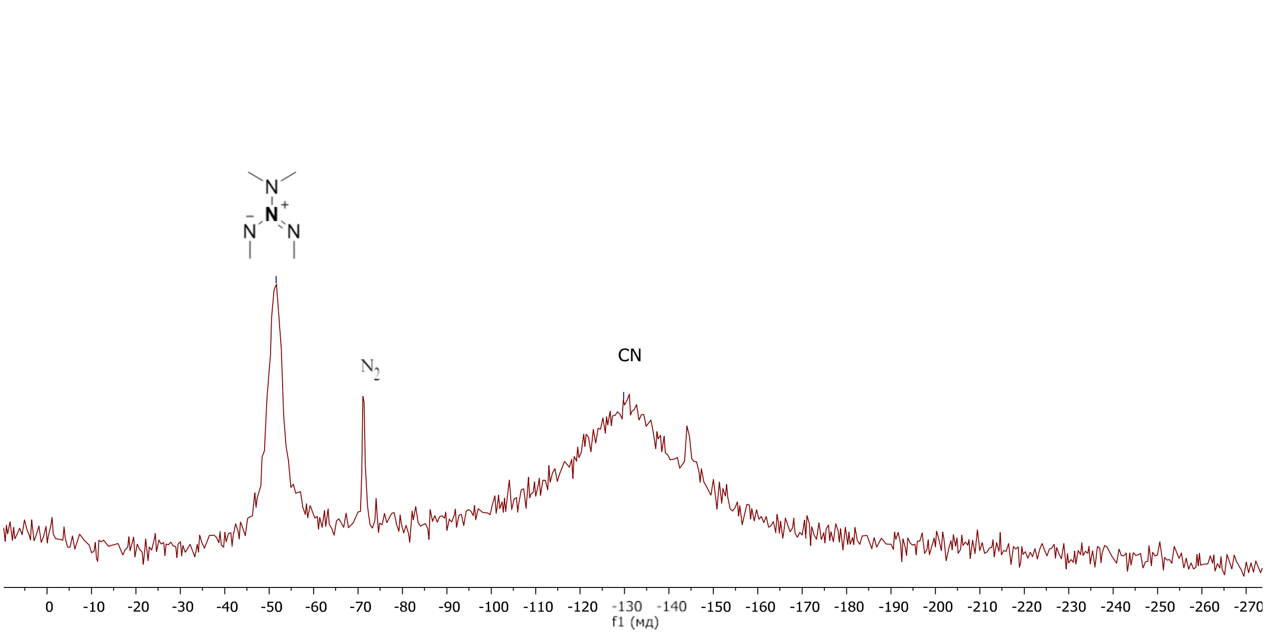

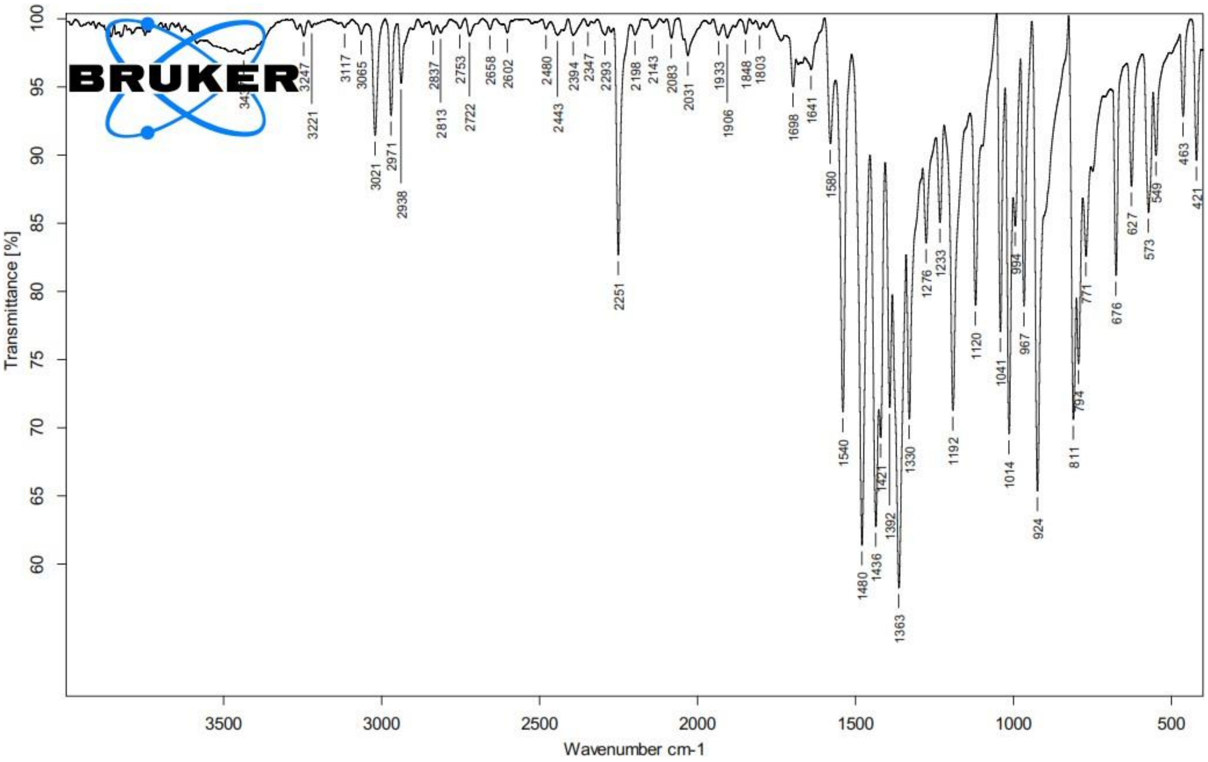

NMR and IR spectra for compound 5

H NMR (600.1 MHz, acetone-d6)

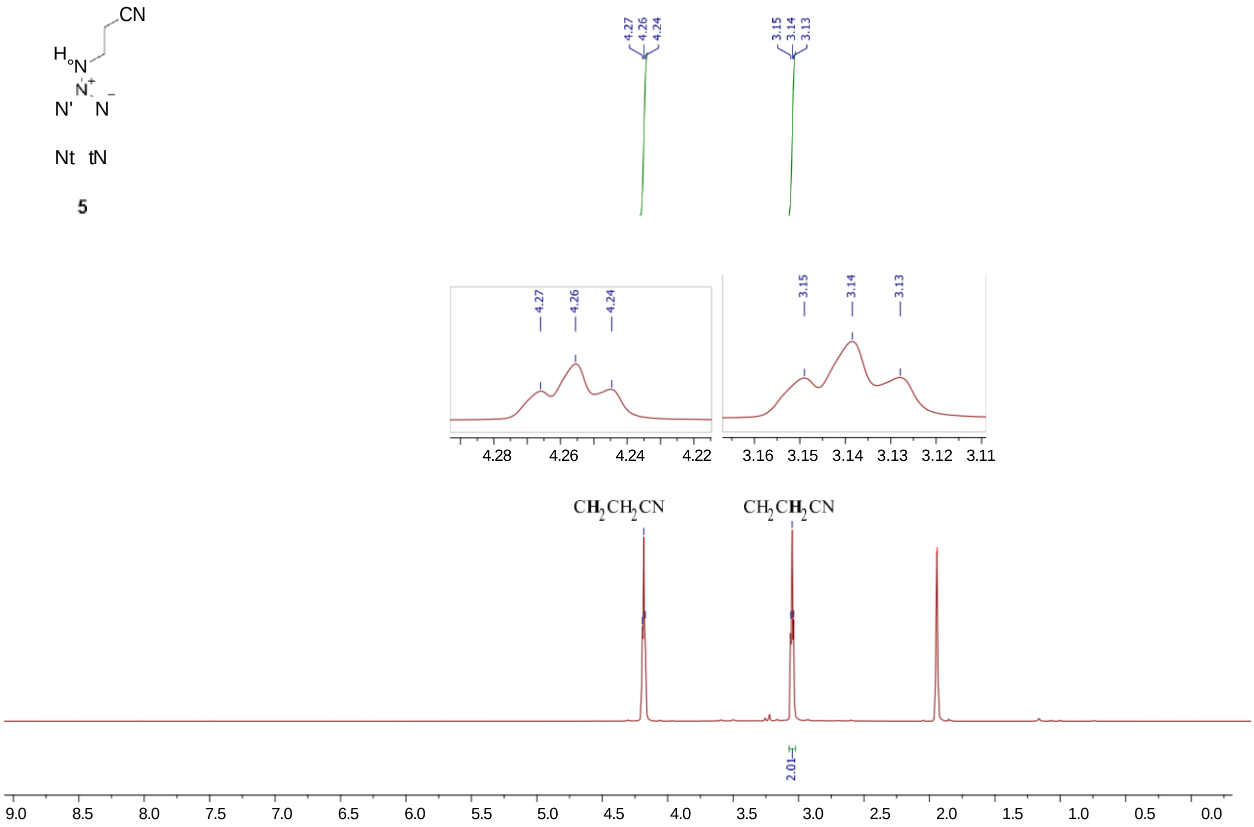

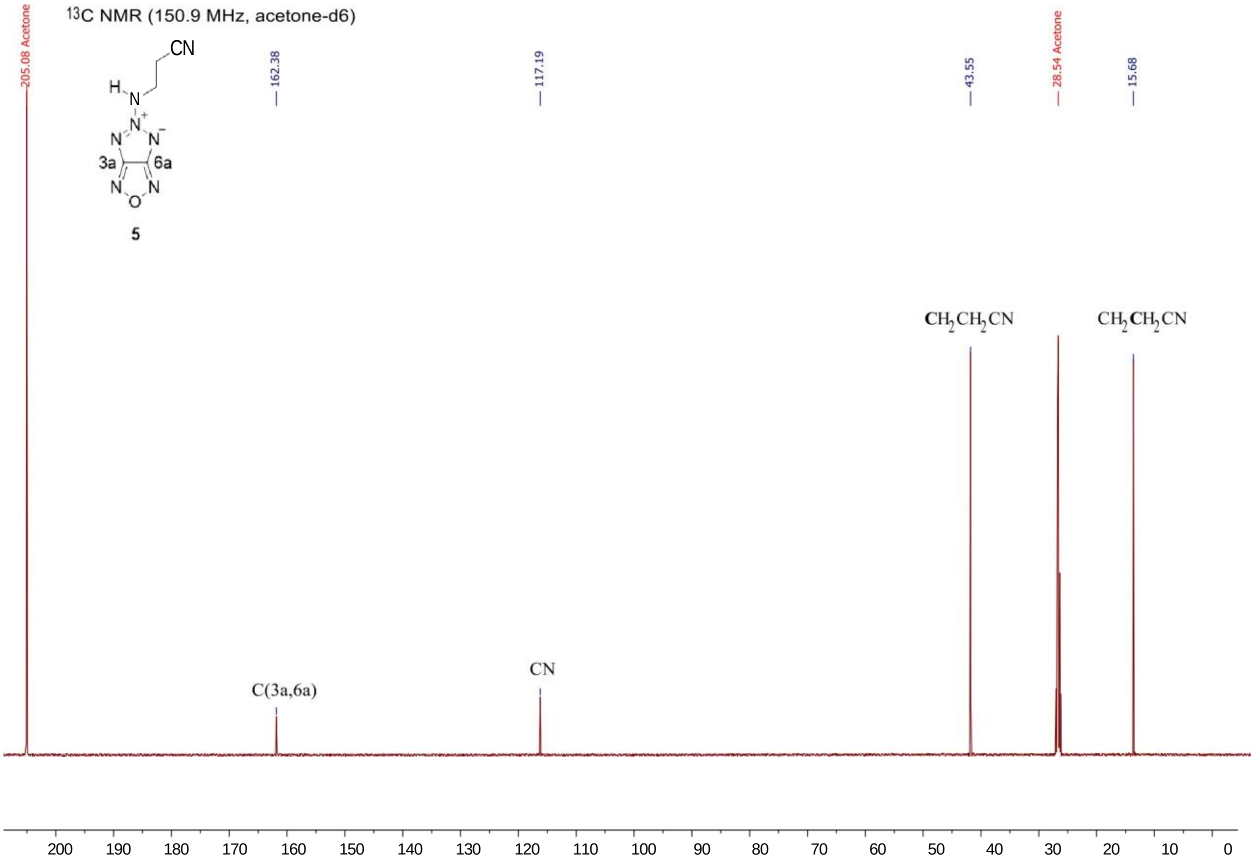

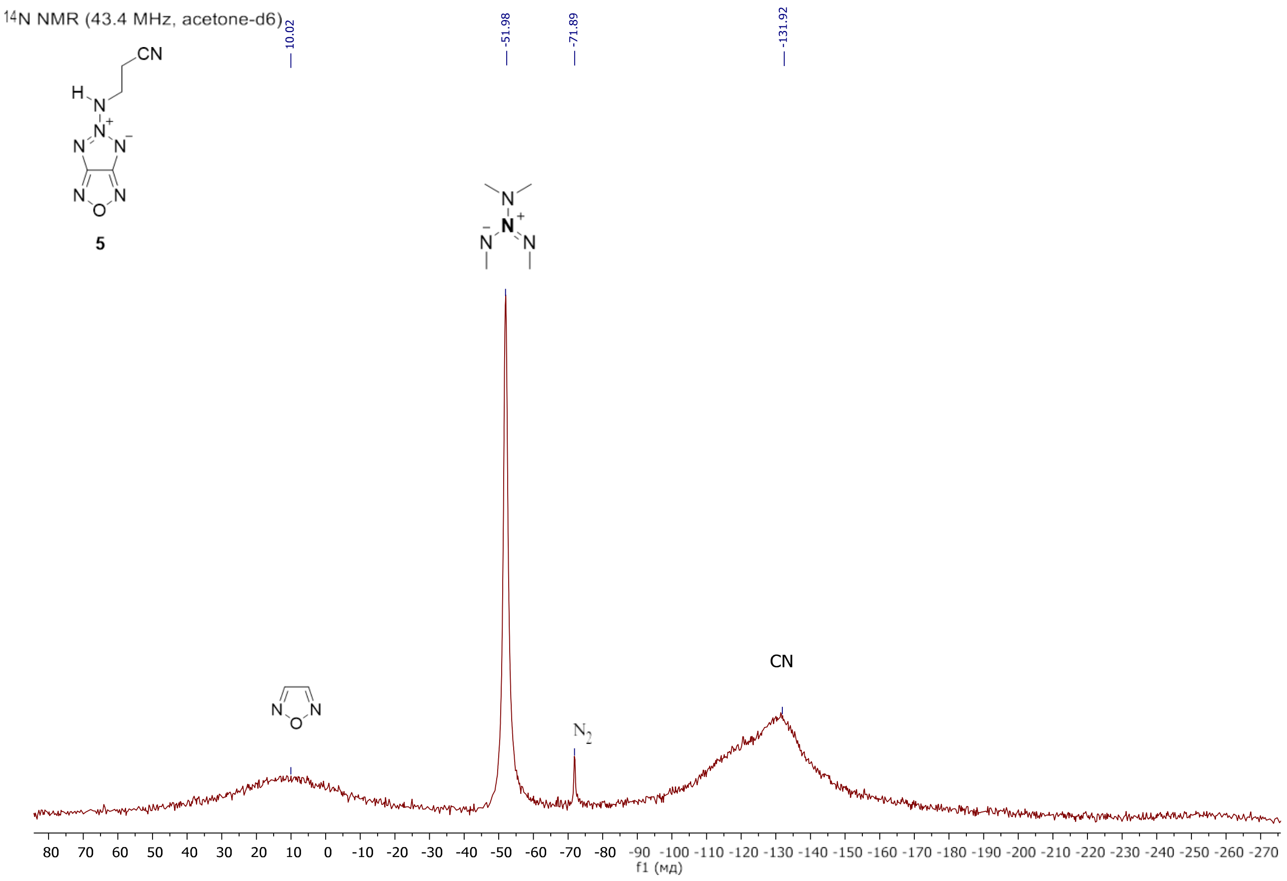
14N NMR (43.4 MHz, acetone-d6)
10.02
-51.98
-71.89
-131.92
CN
5
N2
CN
80 70 60 50 40 30 20 10 0 -10 -20 -30 -40 -50 -60 -70 -80 -90 -100 -110 -120 -130 -140 -150 -160 -170 -180 -190 -200 -210 -220 -230 -240 -250 -260 -270
f1 (мд)

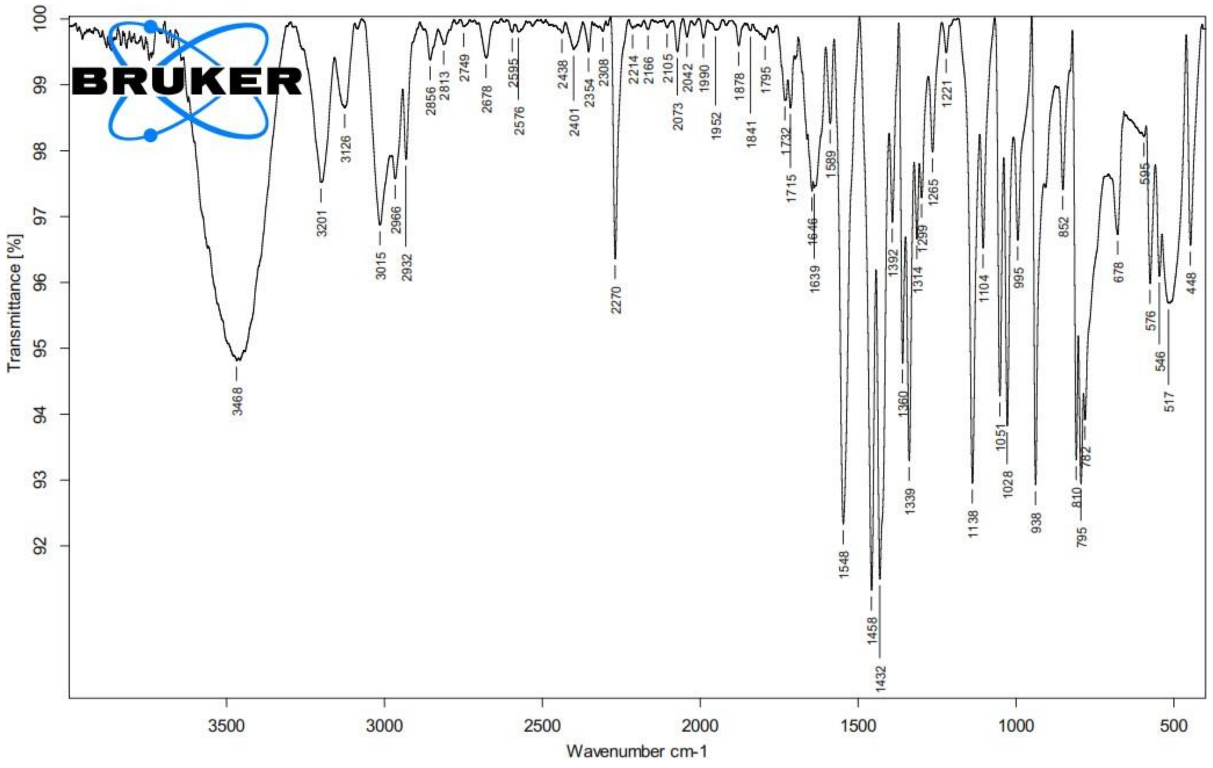
BRUKER
Transmittance [%]
Wavenumber cm-1
3500 3000 2500 2000 1500 1000 500
3468
3201
3126
3015
2966
2932
2856
2813
2749
2678
2595
2576
2438
2401
2354
2308
2270
2214
2166
2105
2073
2042
1990
1952
1878
1841
1795
1732
1715
1646
1639
1589
1548
1458
1432
1392
1360
1339
1314
1299
1265
1221
1138
1104
1051
1028
995
938
852
810
795
782
678
595
576
546
517
448

NMR and IR spectra for compound 4b

H NMR (500.1 MHz, acetone-d6)

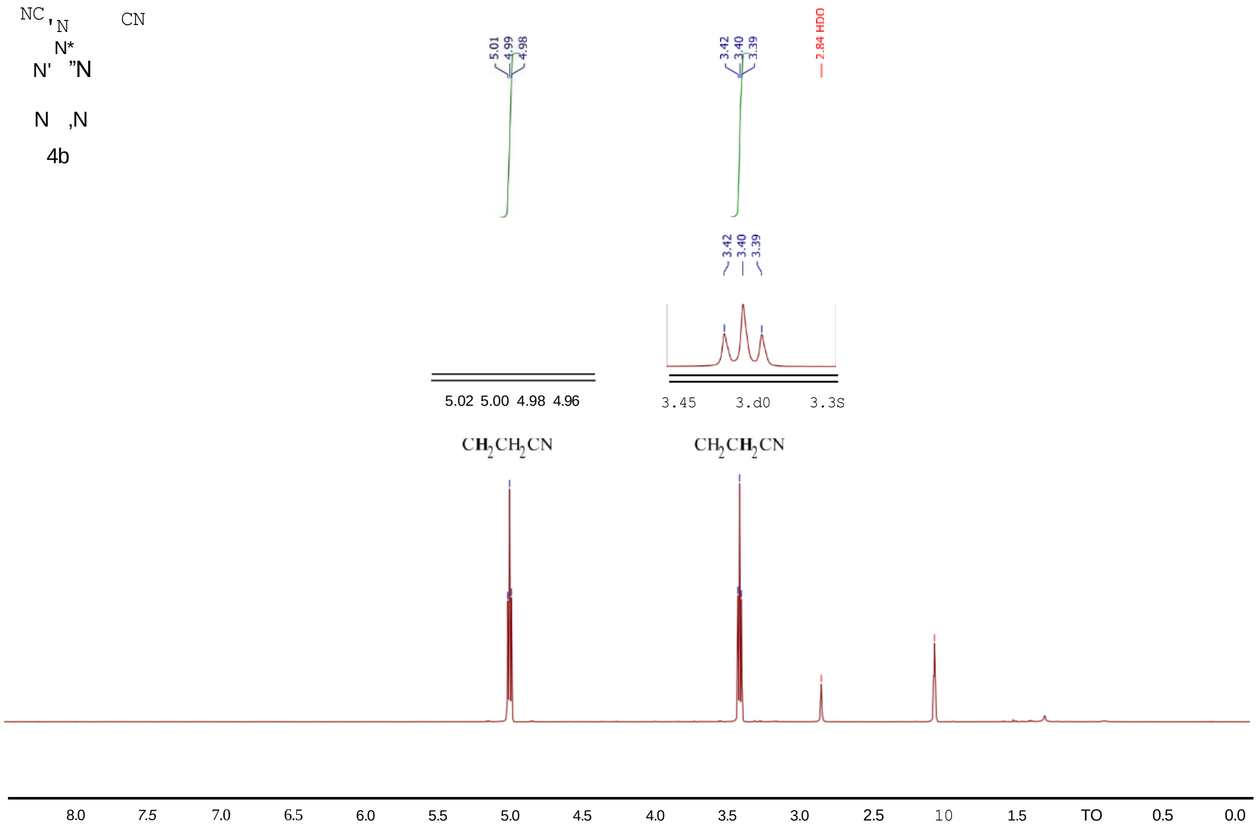

*BC NMR (125 .8 MHz, acetone-d6)

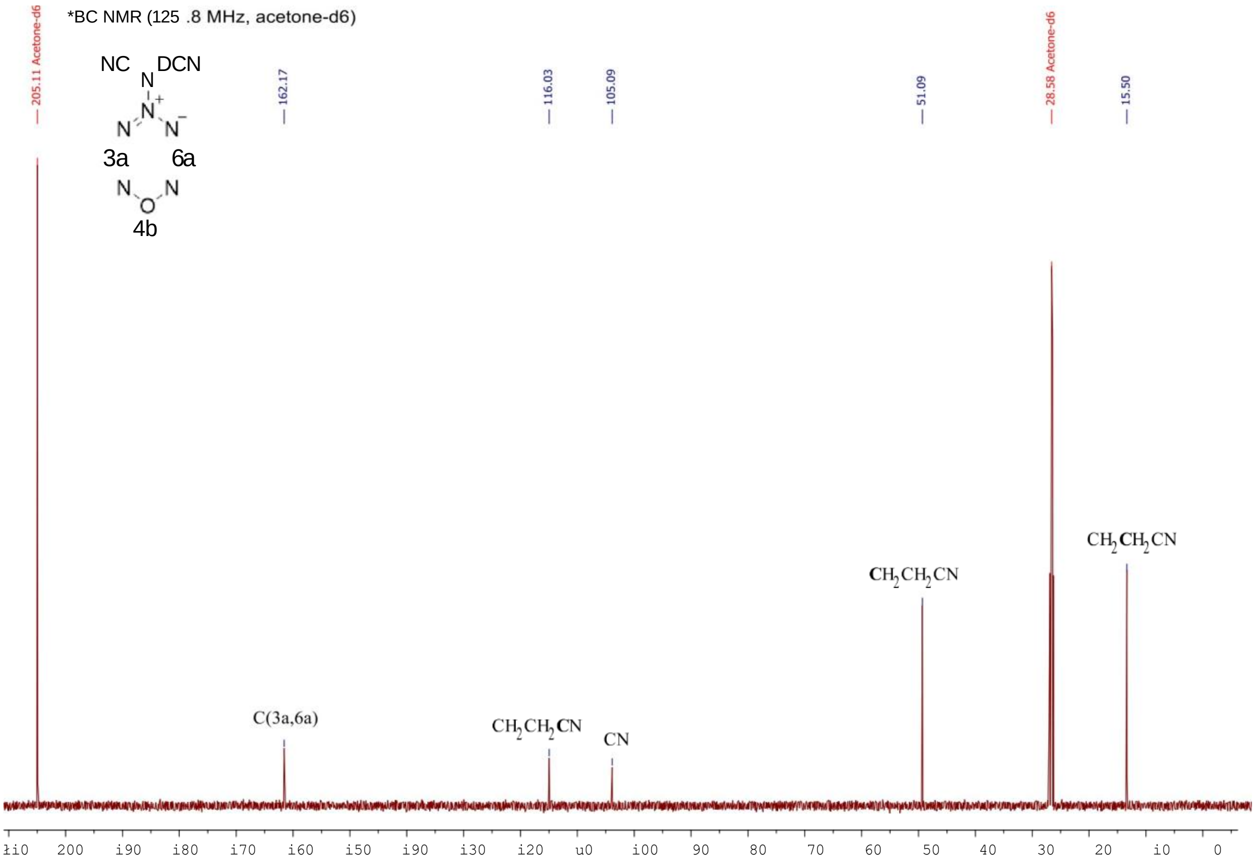

^{14}N NMR (36.1 MHz, acetone-d6)

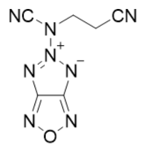

4b

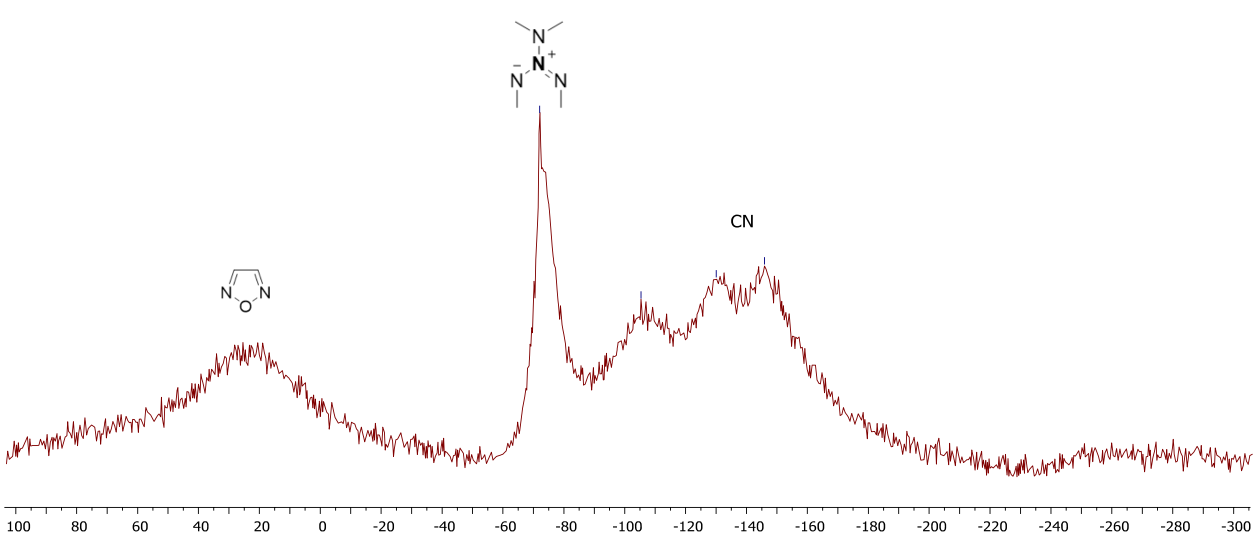

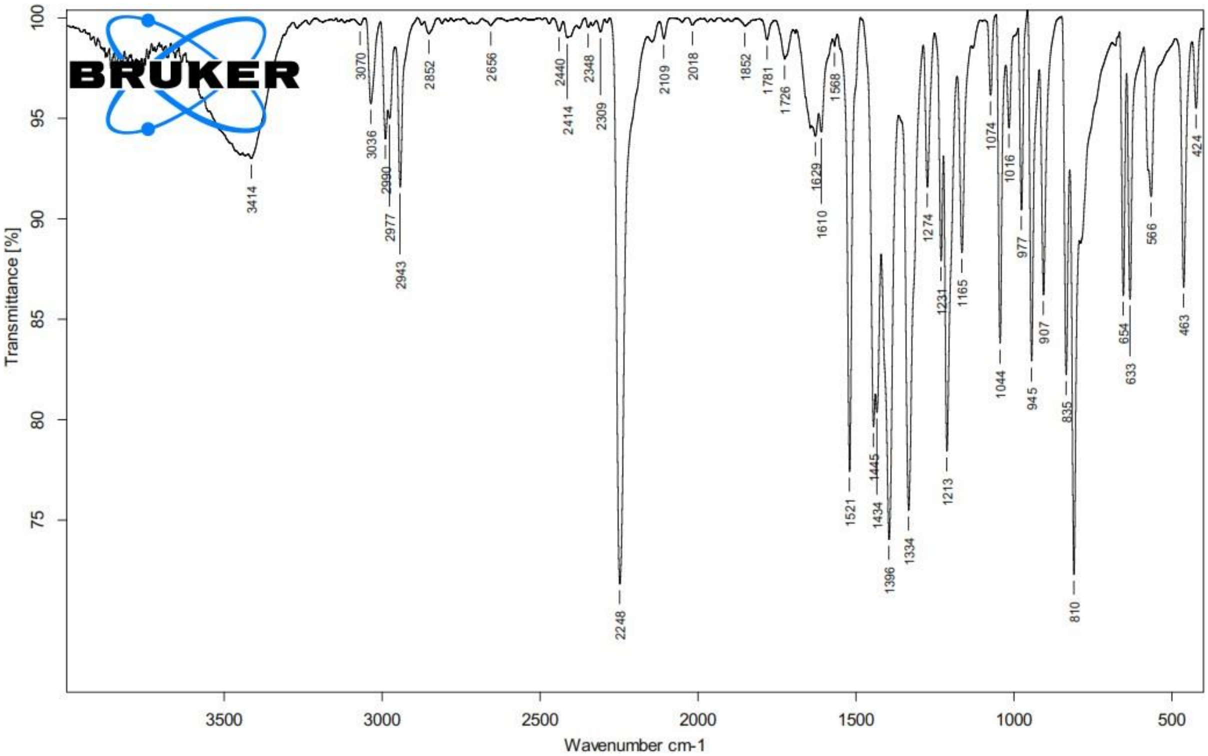

NMR and IR spectra for compound 4c

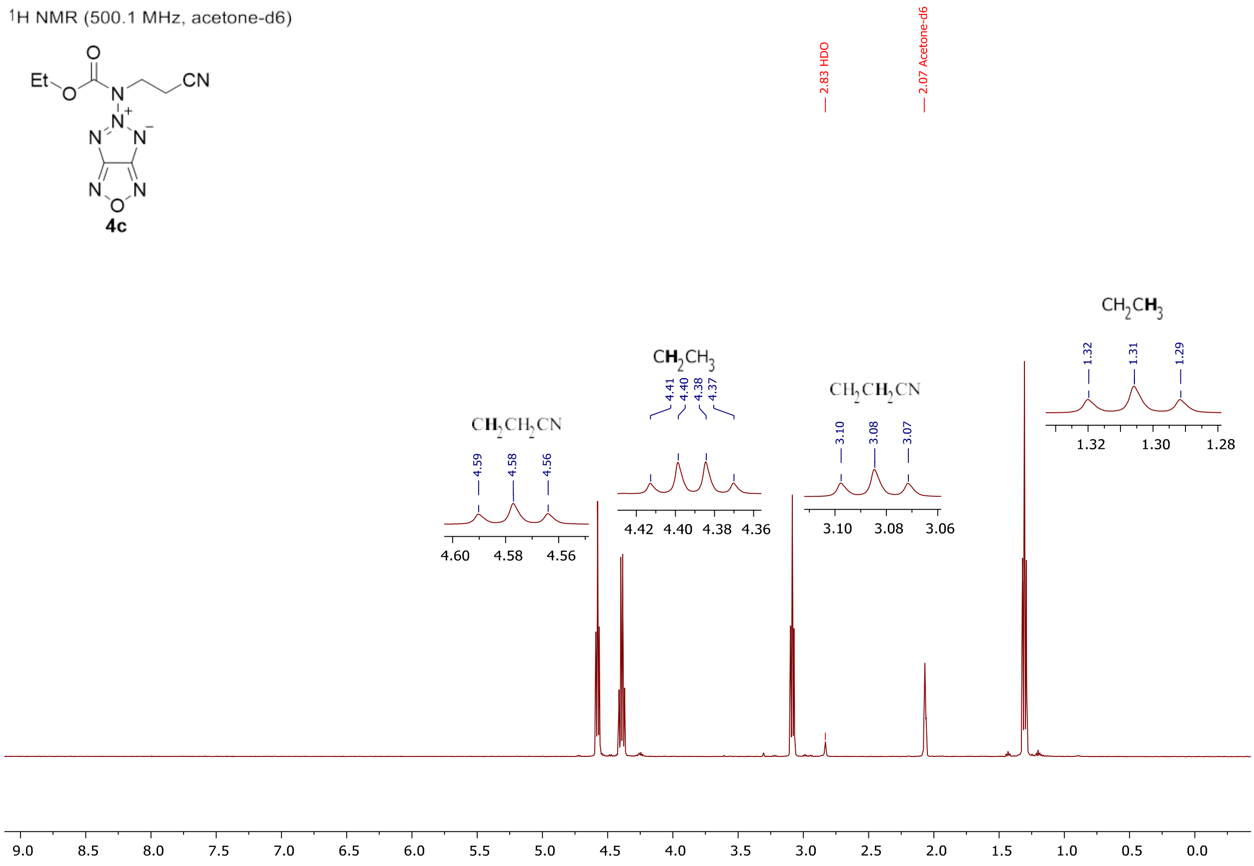

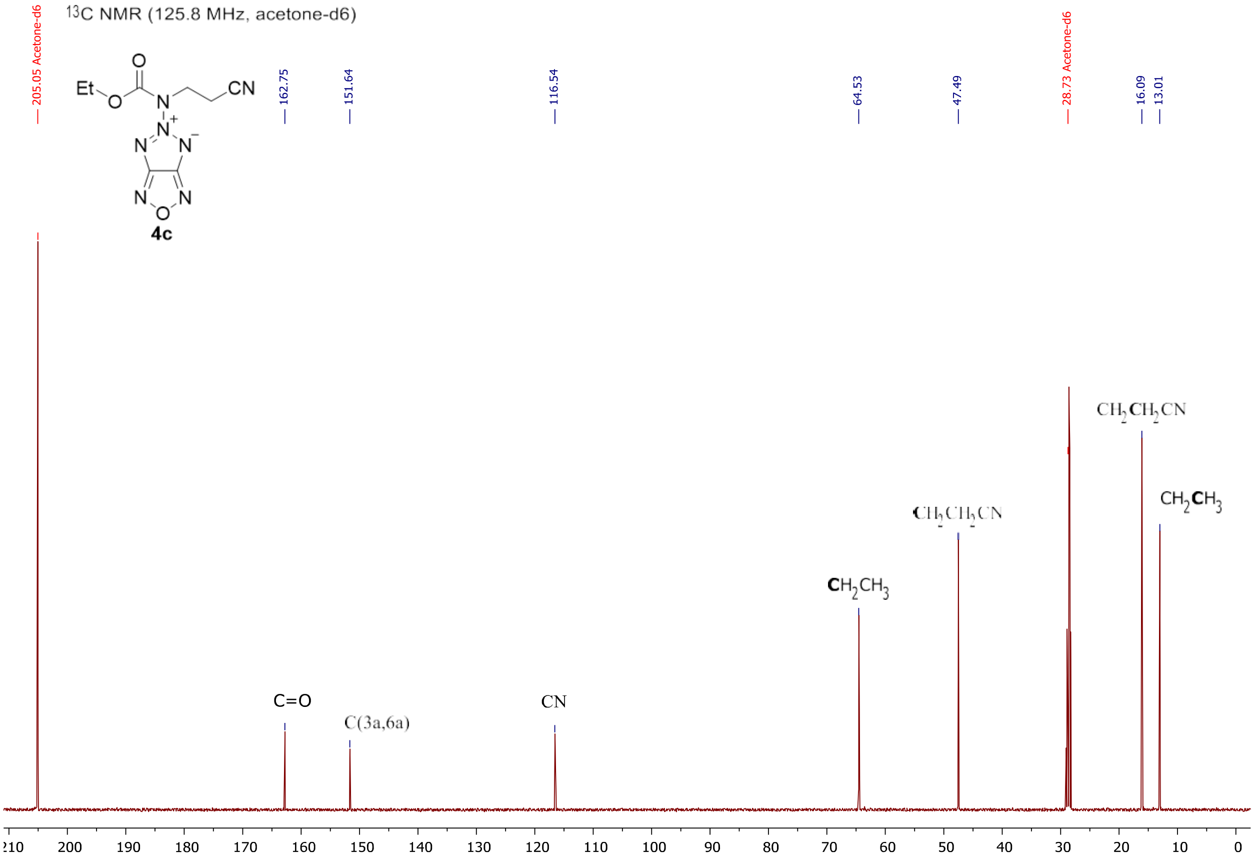

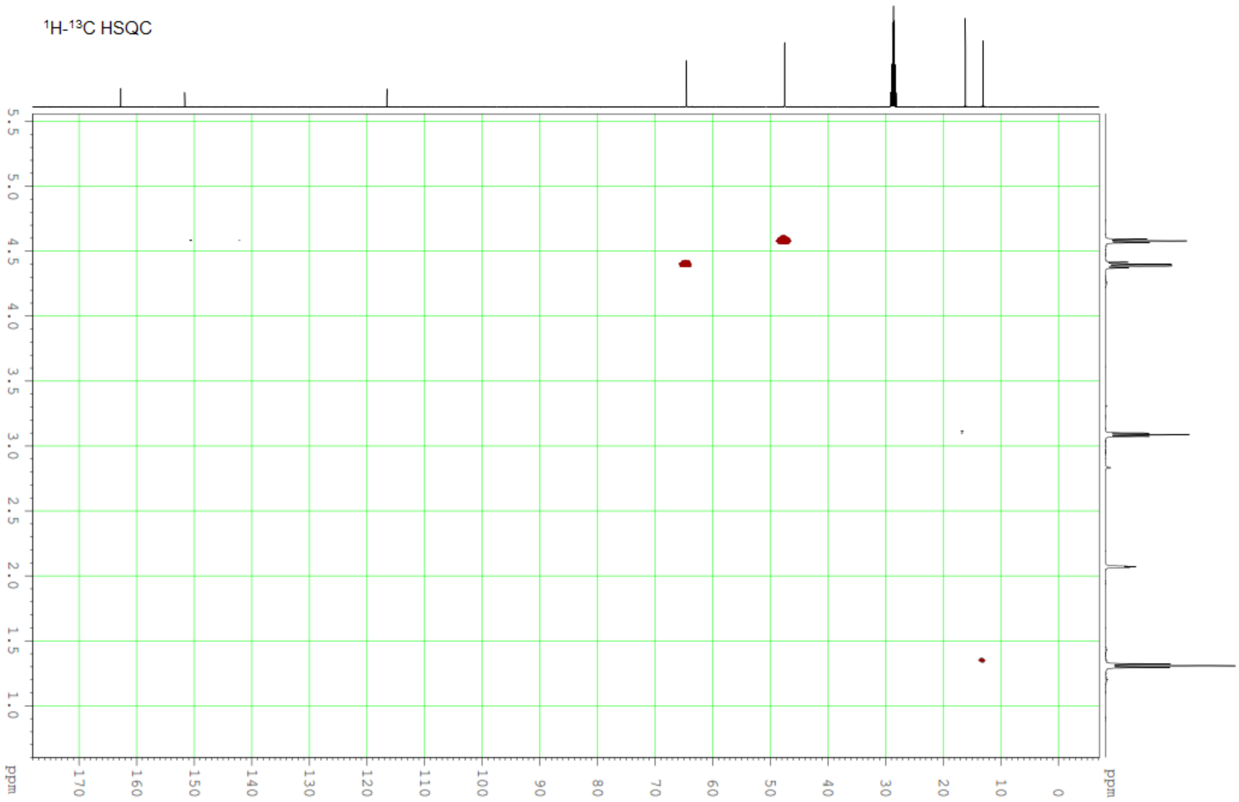

^{1}H-^{13}C HSQC
ppm
170 160 150 140 130 120 110 100 90 80 70 60 50 40 30 20 10 0
5.5 5.0 4.5 4.0 3.5 3.0 2.5 2.0 1.5 1.0

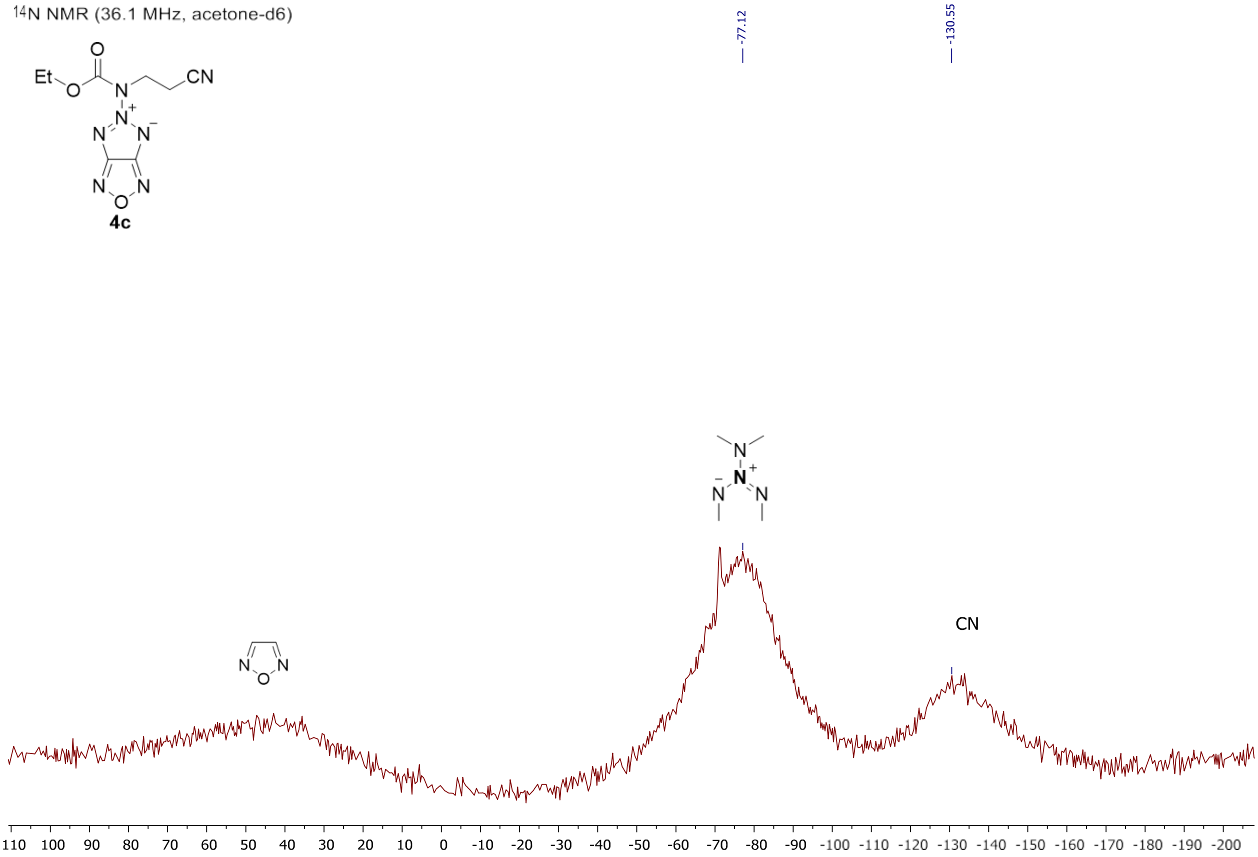

^{14}N NMR (36.1 MHz, acetone-d6)
-77.12
-130.55
4c
CN
110 100 90 80 70 60 50 40 30 20 10 0 -10 -20 -30 -40 -50 -60 -70 -80 -90 -100 -110 -120 -130 -140 -150 -160 -170 -180 -190 -200

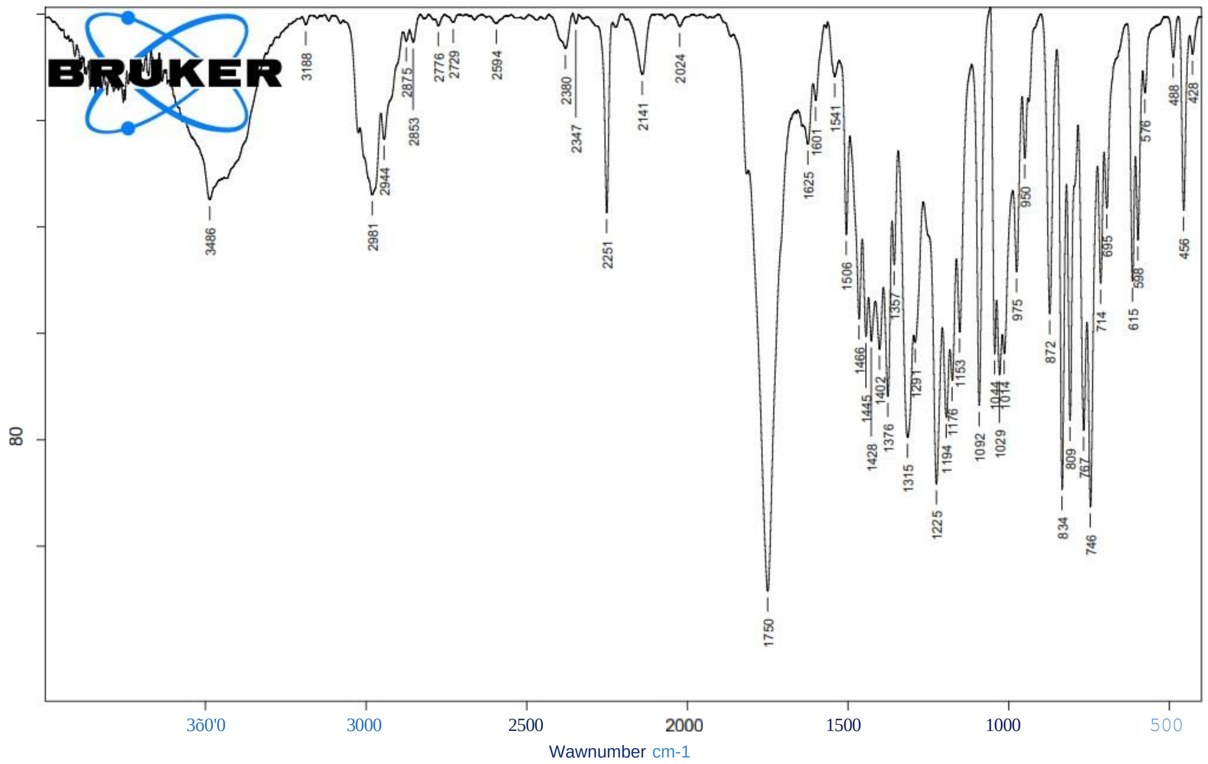
BRUKER
80
3486
3188
2981
2944
2875
2853
2776
2729
2594
2380
2347
2251
2141
2024
1750
1625
1601
1541
1506
1466
1445
1428
1402
1376
1357
1315
1291
1225
1194
1176
1153
1092
1044
1029
1014
975
950
872
834
809
767
746
714
695
615
598
576
488
456
428
3000
2500
2000
1500
1000
500
Wawnumber cm-1

NMR and IR spectra for compound 3a

BC NMR (125.8 MHz, DMSO-d6)

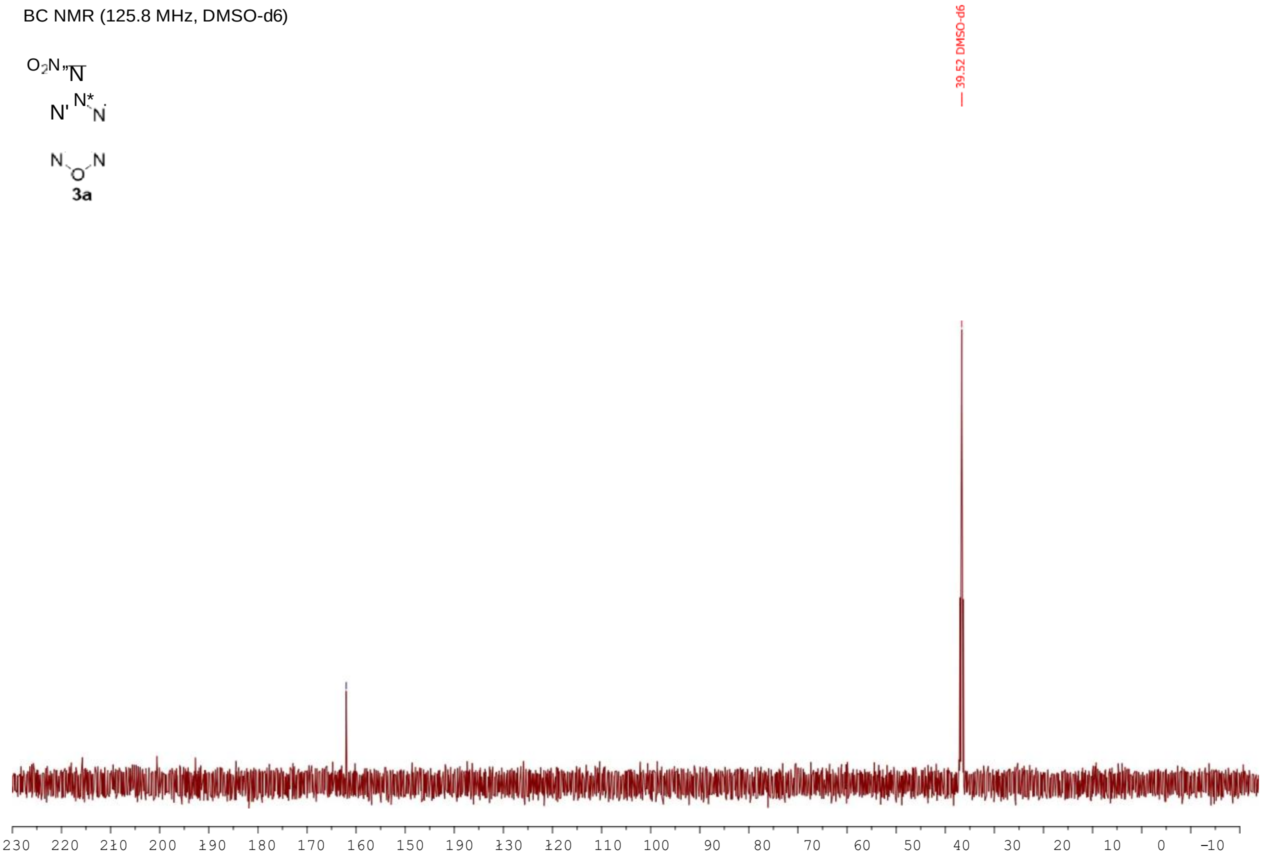

14N NMR (36.1 MHz, DMSO-d6)

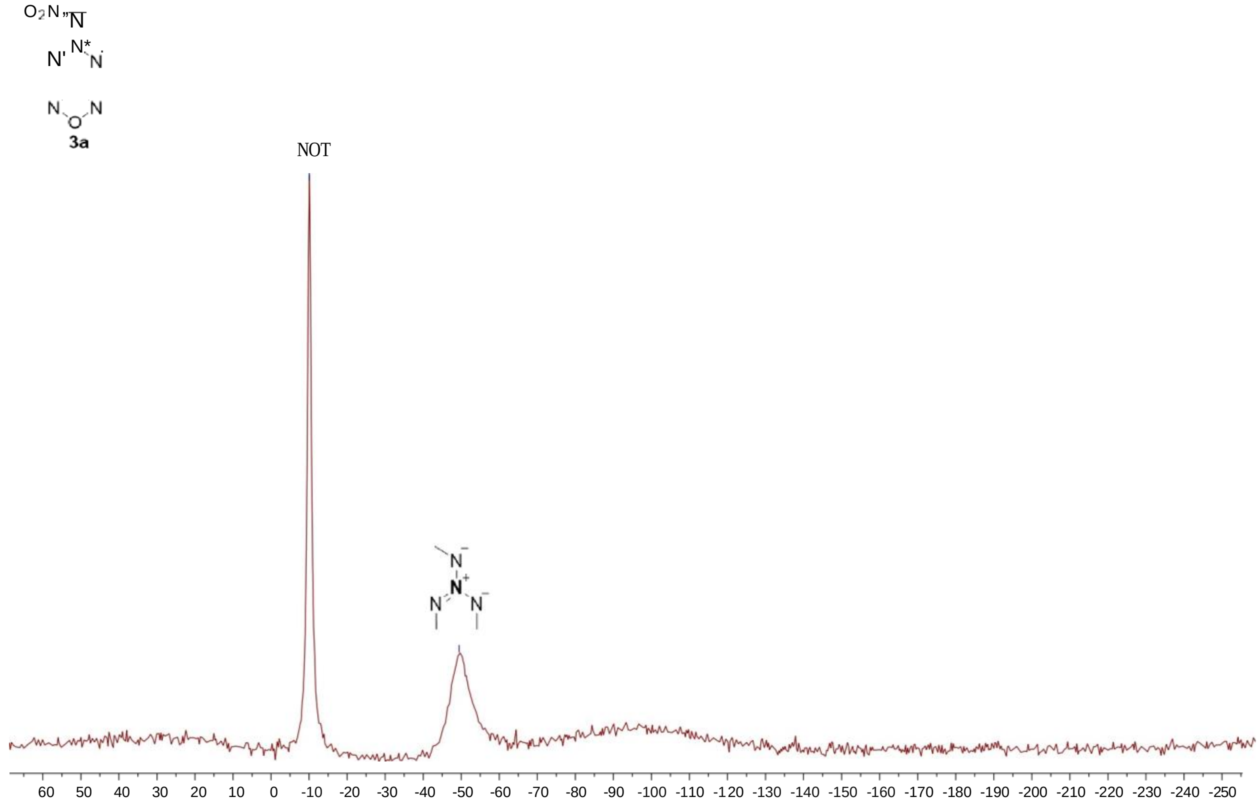

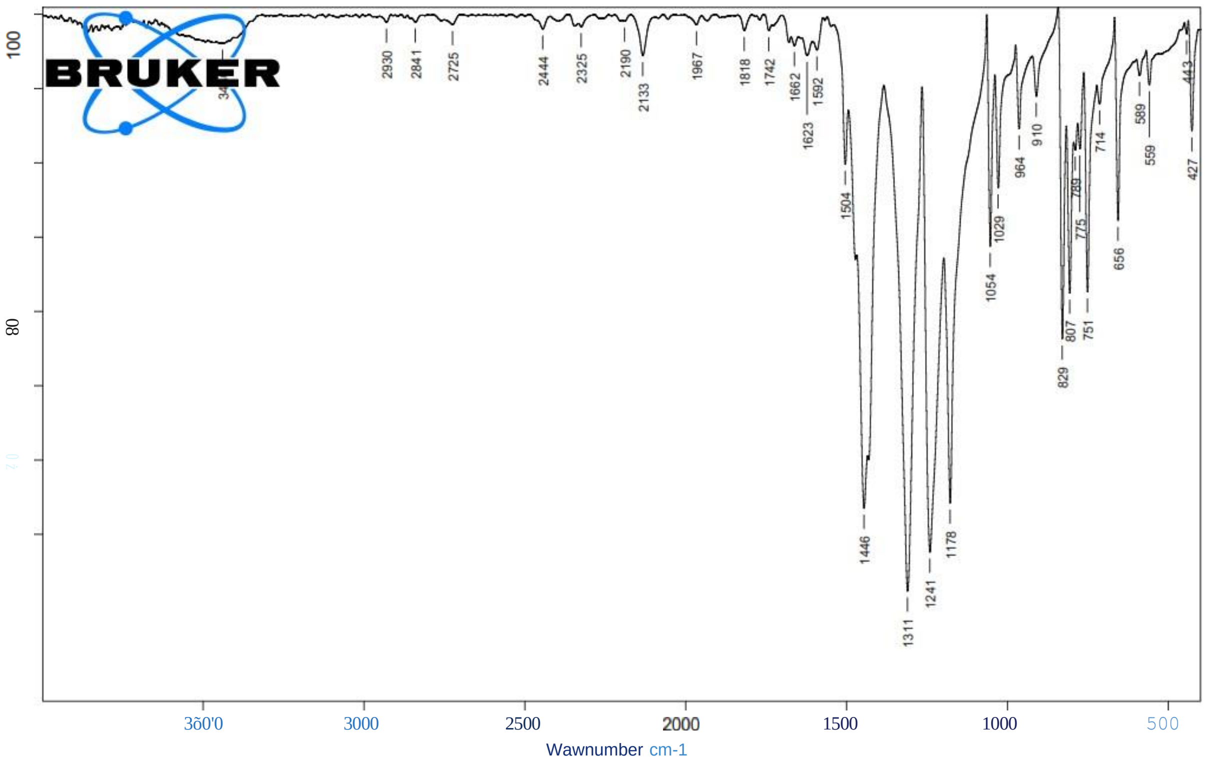
BRUKER
100
80
2930
2841
2725
2444
2325
2190
2133
1967
1818
1742
1662
1623
1592
1504
1446
1311
1241
1178
1054
1029
964
910
829
807
789
775
751
714
656
589
559
443
427
3000
2500
2000
1500
1000
500
Wawnumber cm-1

NMR spectra for compound 3a – MeCN complex

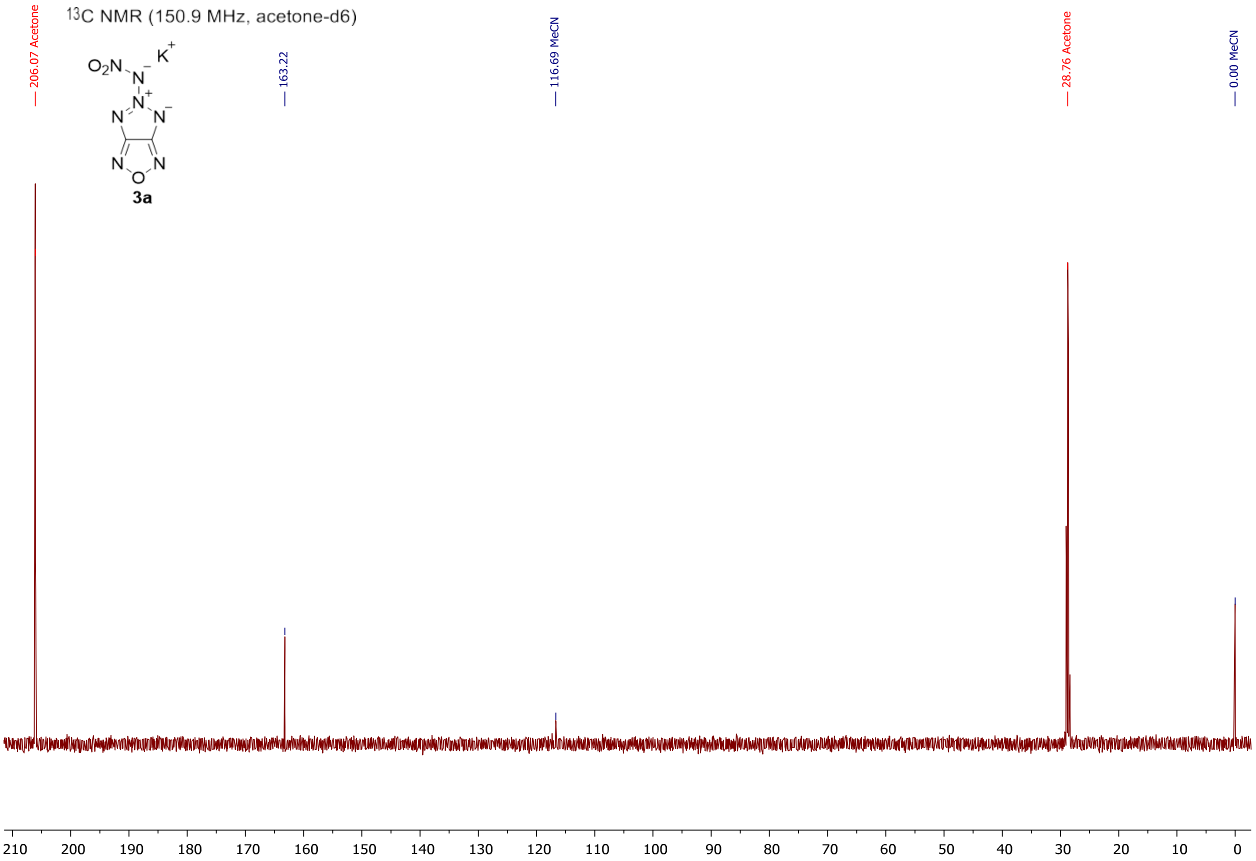

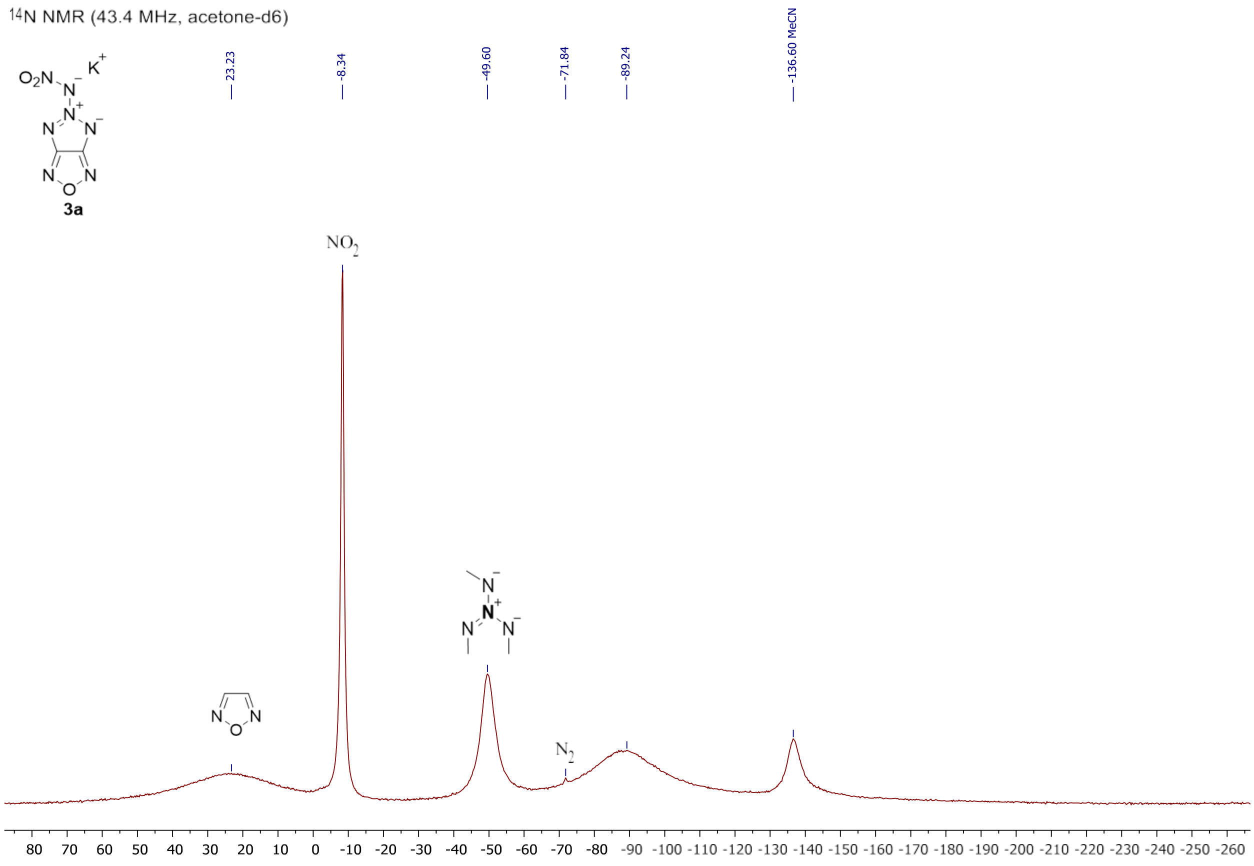

^{15}N NMR (60.8 MHz, acetone-d6)

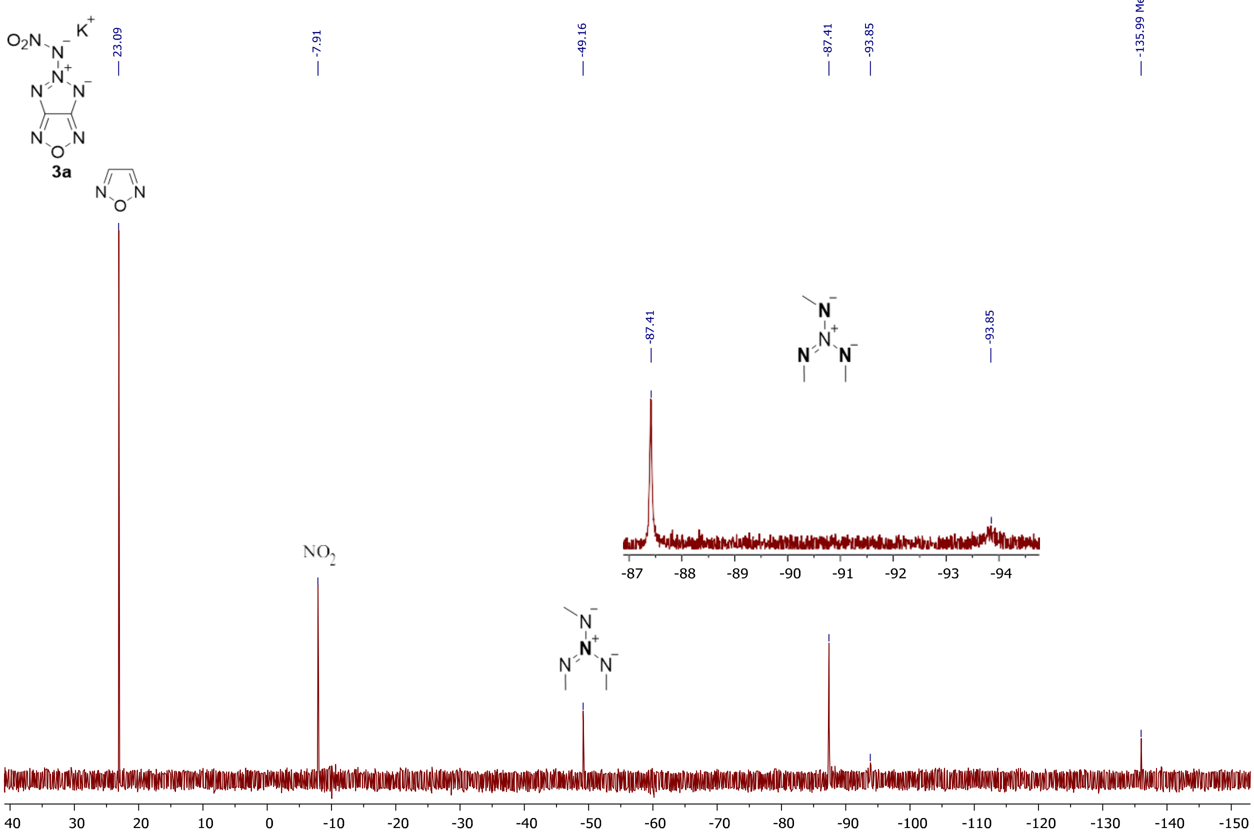

NMR and IR spectra for compound 3b

C NMR (150.9 MHz, methanol-d4)

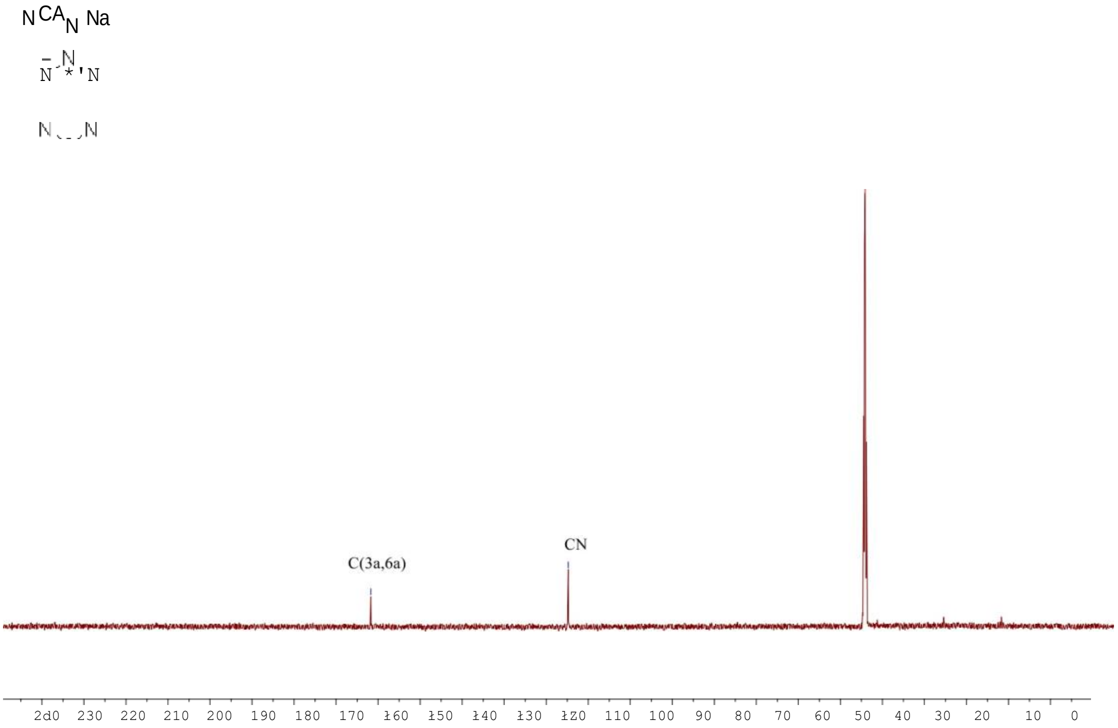

14N NMR (36.1 MHz, methanol-d4)

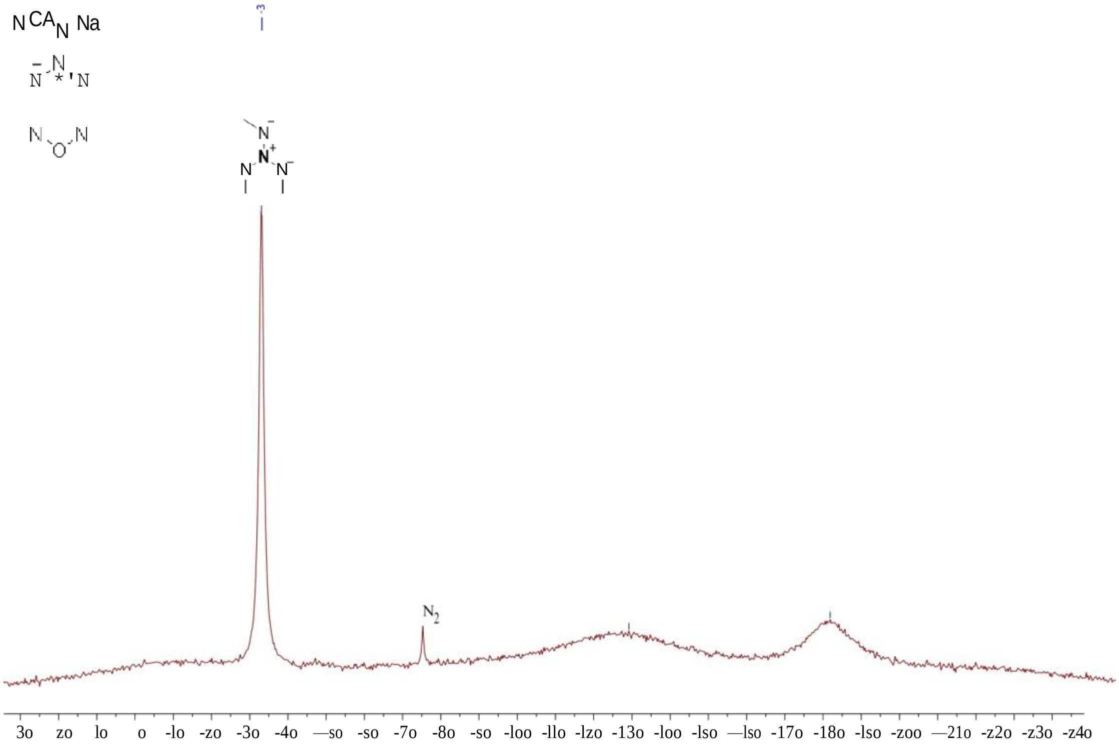

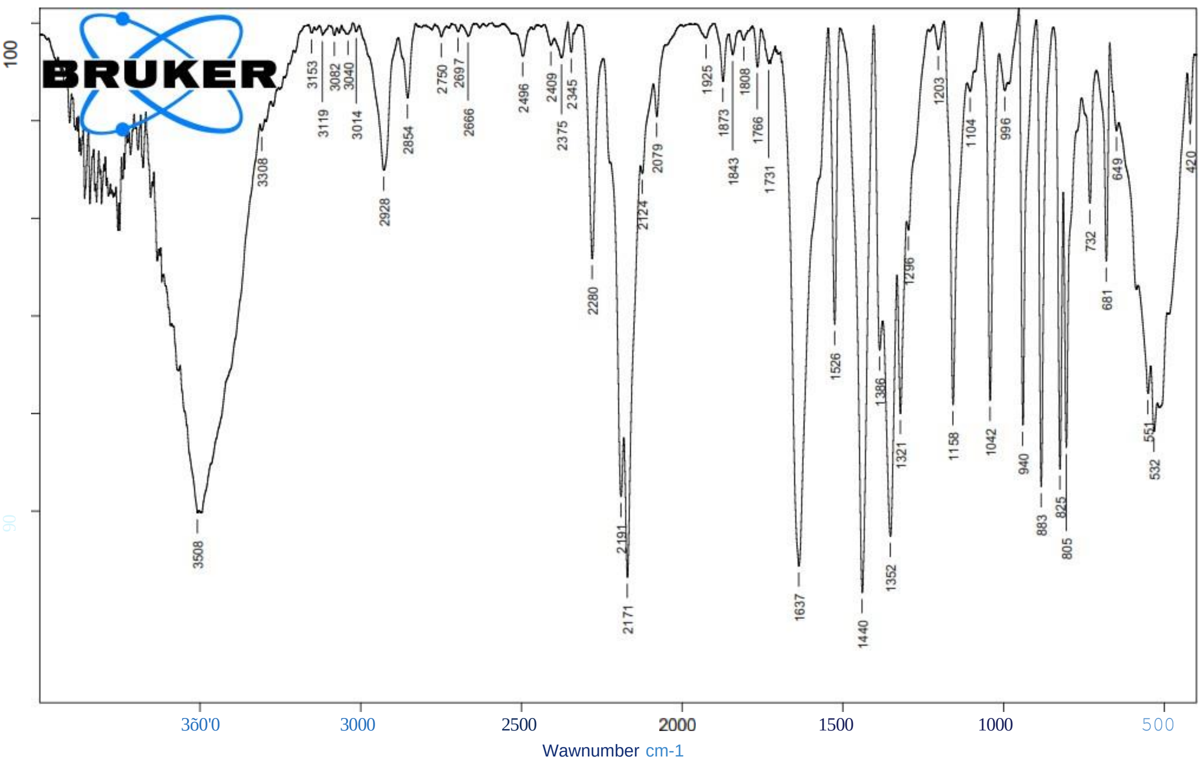

BRUKER
100
90
3500
3000
2500
2000
1500
1000
500
Wawnumber cm-1

NMR and IR spectra for compound 3c

H NMR (600.1 MHz, DMSO-d6)

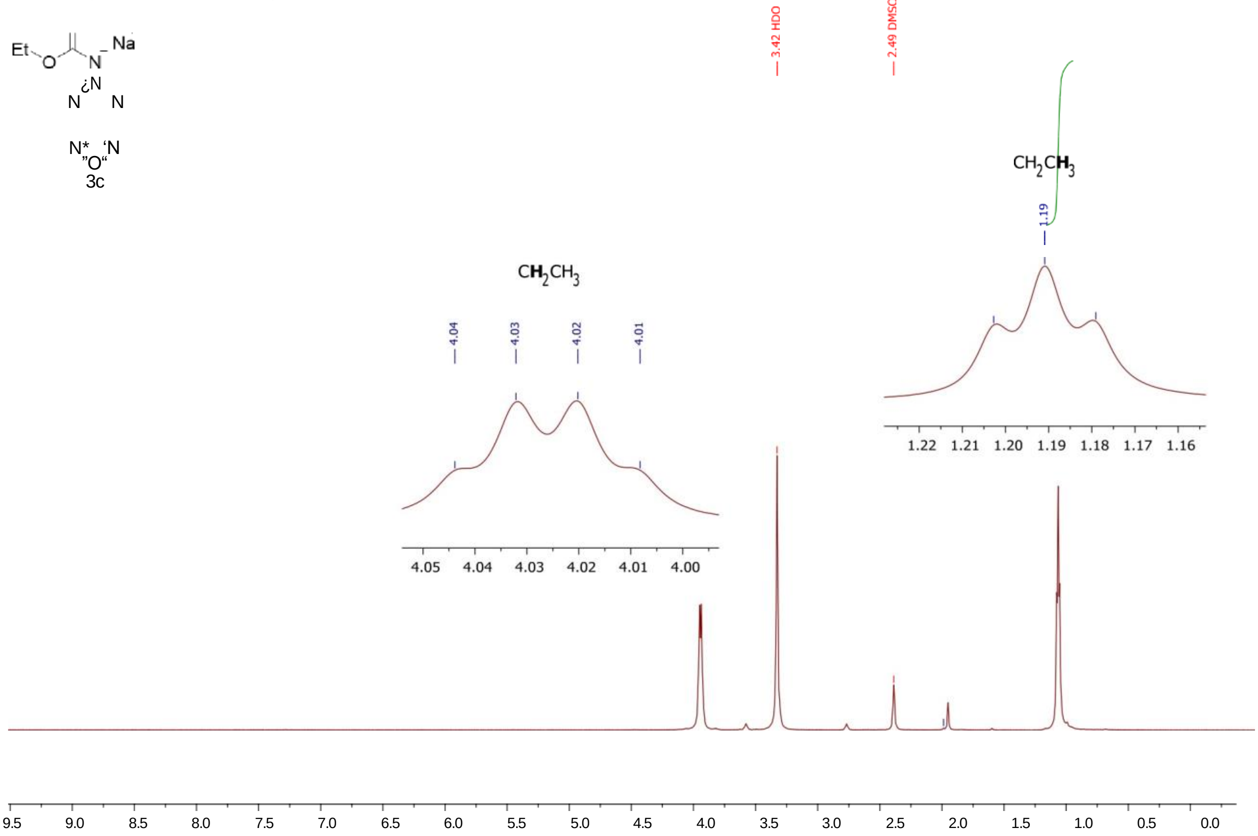

BC NMR (150.9 MHz, DMSO-d6)

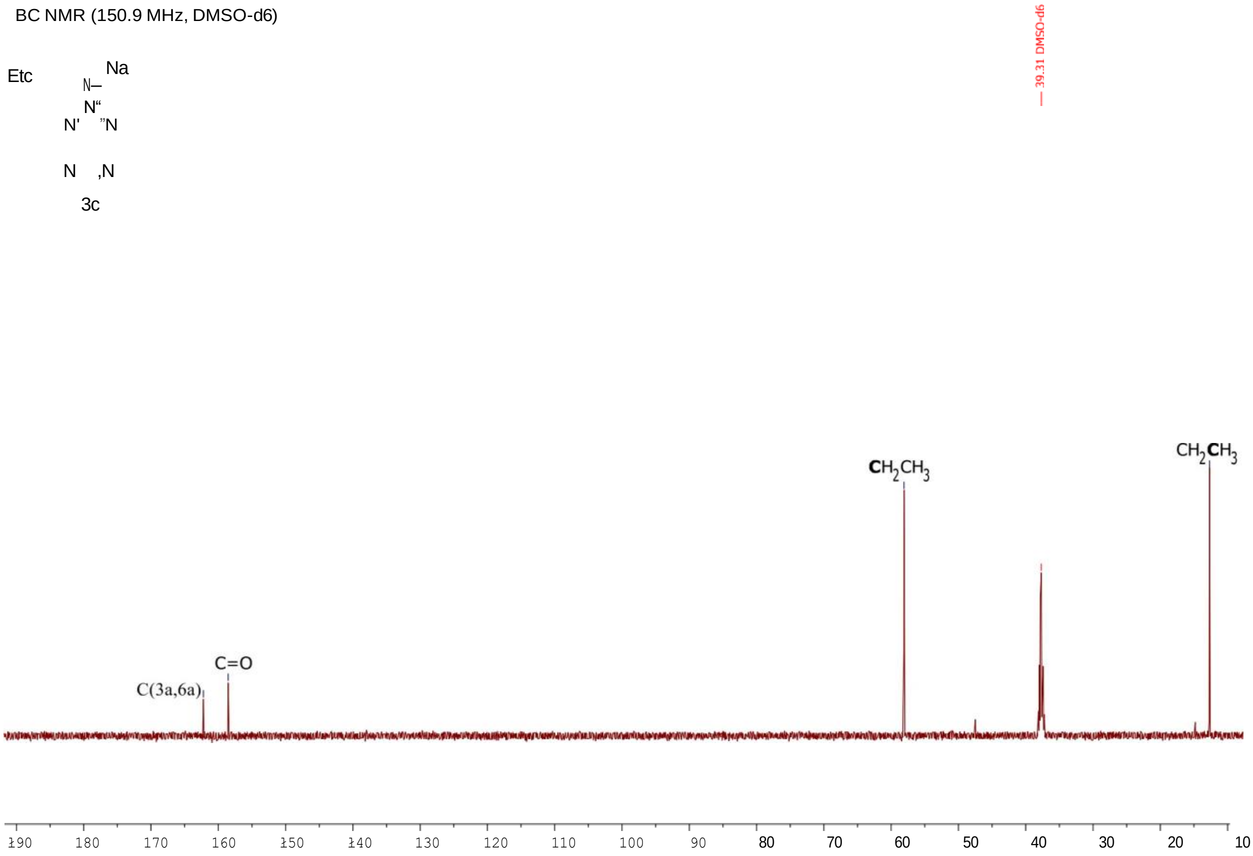

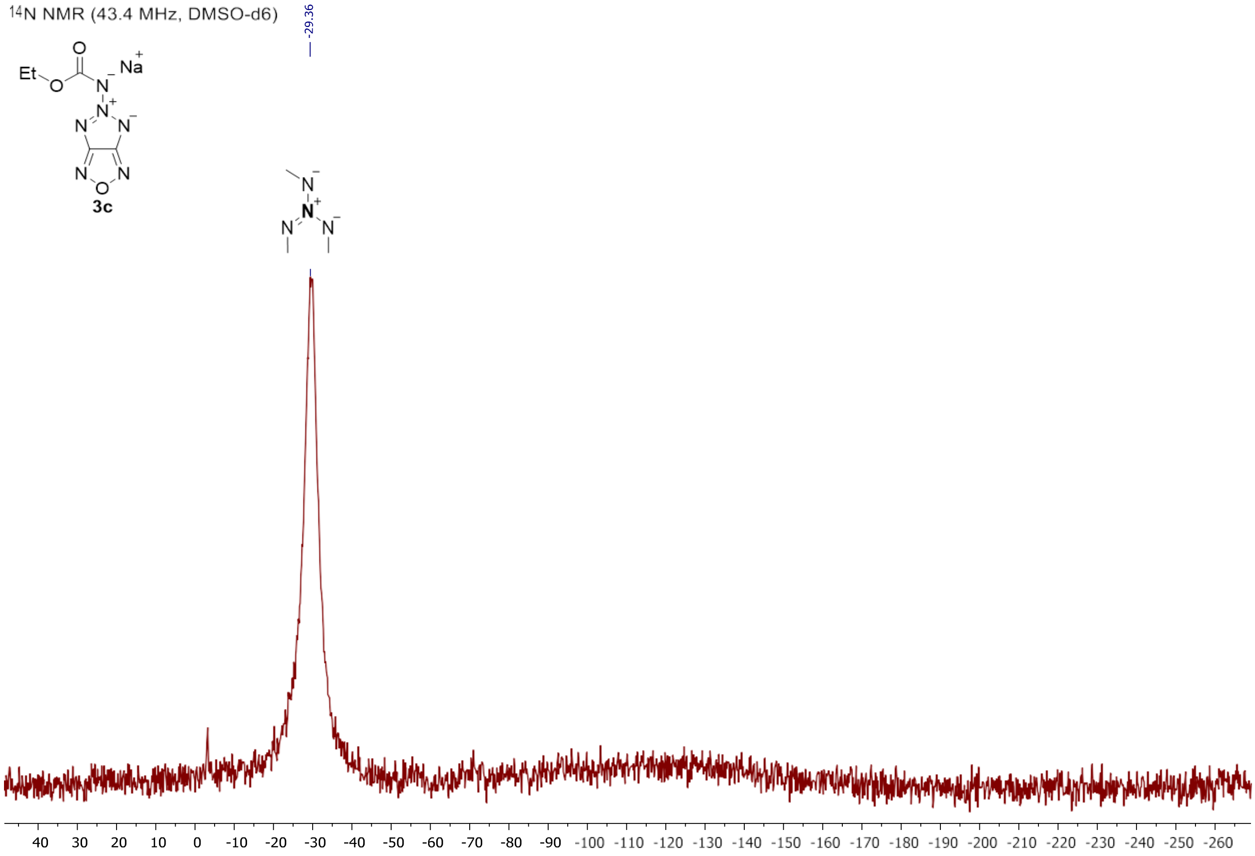

14N NMR (43.4 MHz, DMSO-d6)
-29.36
Et
O
N
Na+
3c
40 30 20 10 0 -10 -20 -30 -40 -50 -60 -70 -80 -90 -100 -110 -120 -130 -140 -150 -160 -170 -180 -190 -200 -210 -220 -230 -240 -250 -260

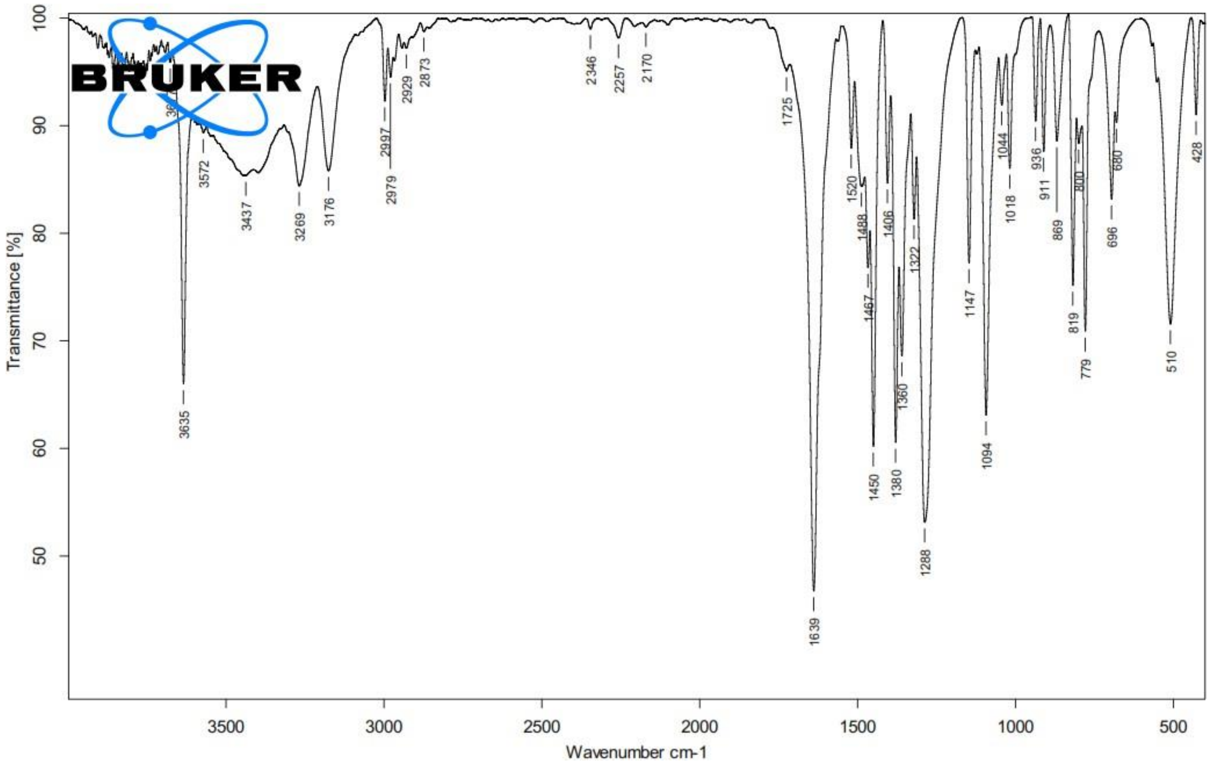

BRUKER
Transmittance [%]
Wavenumber cm-1
100
90
80
70
60
50
3500 3000 2500 2000 1500 1000 500
3635
3572
3437
3269
3176
2997
2979
2929
2873
2346
2257
2170
1725
1639
1520
1488
1467
1450
1406
1380
1360
1322
1288
1147
1094
1044
1018
936
911
869
819
800
779
696
680
510
428
